# Patterns and predictors of alcohol misuse trajectories from adolescence through early midlife

**DOI:** 10.1017/S0954579424000543

**Published:** 2024-03-11

**Authors:** Mallory Stephenson, Peter Barr, Nathaniel Thomas, Megan Cooke, Antti Latvala, Richard J. Rose, Jaakko Kaprio, Danielle Dick, Jessica E. Salvatore

**Affiliations:** 1 Virginia Institute for Psychiatric and Behavioral Genetics, Virginia Commonwealth University, Richmond, VA, USA; 2 Department of Psychiatry and Behavioral Sciences, SUNY Downstate Health Sciences University, Brooklyn, NY, USA; 3 Department of Psychology, Virginia Commonwealth University, Richmond, VA, USA; 4 Department of Psychiatry, Robert Wood Johnson Medical School, Rutgers University, New Brunswick, NJ, USA; 5 Institute of Criminology and Legal Policy, University of Helsinki, Helsinki, Finland; 6 Department of Psychological and Brain Sciences, Indiana University, Bloomington, IN, USA; 7 Department of Public Health, Institute for Molecular Medicine Finland, University of Helsinki, Helsinki, Finland

**Keywords:** alcohol, biometric, early midlife, genetic, growth curve, trajectories

## Abstract

We took a multilevel developmental contextual approach and characterized trajectories of alcohol misuse from adolescence through early midlife, examined genetic and environmental contributions to individual differences in those trajectories, and identified adolescent and young adult factors associated with change in alcohol misuse. Data were from two longitudinal population-based studies. FinnTwin16 is a study of Finnish twins assessed at 16, 17, 18, 25, and 35 years (N=5659; 52% female; 32% monozygotic). The National Longitudinal Study of Adolescent to Adult Health (Add Health) is a study of adolescents from the United States, who were assessed at five time points from 1994 to 2018 (N=18026; 50% female; 64% White, 21% Black, 4% Native American, 7% Asian, 9% Other race/ethnicity). Alcohol misuse was measured as frequency of intoxication in FinnTwin16 and frequency of binge drinking in Add Health. In both samples, trajectories of alcohol misuse were best described by a quadratic growth curve: Alcohol misuse increased across adolescence, peaked in young adulthood, and declined into early midlife. Individual differences in these trajectories were primarily explained by environmental factors. Several adolescent and young adult correlates were related to the course of alcohol misuse, including other substance use, physical and mental health, and parenthood.

Alcohol misuse, which includes patterns of binge drinking (blood alcohol concentration greater than 0.08%) and heavy drinking (7+ drinks per week for women and 14+ drinks for men) (National Institute on Alcohol Abuse and Alcoholism, n.d.), is a significant public health concern. Even moderate levels of alcohol consumption are associated with poorer physical health (Cho et al., 2015; Holmes et al., 2014; Wood et al., 2018), lower neurocognitive performance (Topiwala et al., 2022), and increased mortality (Hublin & Kaprio, 2022), and heavy drinking further elevates risk for morbidity and mortality (Hublin & Kaprio, 2022; Plunk et al., 2014; Roerecke & Rehm, 2010).

The vast majority of longitudinal studies on the development of alcohol misuse focus on adolescence through young adulthood, perhaps driven by the idea that many individuals “mature out” of binge drinking during the transition to adulthood (Lee & Sher, 2018). Nonetheless, alcohol misuse remains common in early midlife, with 32% of individuals in their 30s and 40s reporting past-month binge drinking (Substance Abuse and Mental Health Services Administration, 2018). Further, middle-aged Americans have uniquely experienced an increase in all-cause mortality over the past 20 years. Rising mortality rates in this age group are primarily driven by suicide, drug and alcohol poisoning, and chronic liver diseases and cirrhosis (Case & Deaton, 2015), all of which are directly or indirectly linked to alcohol misuse (Brady, 2006; Rehm et al., 2010). Therefore, delineating the nature and predictors of alcohol misuse trajectories through early midlife may provide critical insight into morbidity and mortality and highlight targets for prevention and intervention efforts.

Previous efforts to describe trajectories of alcohol misuse through early midlife have primarily relied upon person-centered approaches (Berg et al., 2013, 2019; Jackson & Sher, 2005; Vachon et al., 2017; Virtanen et al., 2015), which classify individuals into groups based on their patterns of change in alcohol misuse (Jung & Wickrama, 2008). These methods are appealing because they may identify distinct subpopulations of individuals at particular risk for alcohol problems (Muthén & Muthén, 2000). However, person-based trajectory approaches nearly always yield four groups, which are characterized by consistently low levels of alcohol involvement, increasing alcohol involvement across the study period, decreasing alcohol involvement across the study period, and consistently high levels of alcohol involvement. Prior work suggests that identification of these four typologies may be driven by the analytic approach, rather than meaningful variation in alcohol misuse (Sher et al., 2011). Therefore, variable-centered approaches, which focus on the relationships among variables rather than the relationships among individuals, are also needed to characterize developmental trajectories of alcohol misuse.

Several studies have adopted a variable-centered approach to describe trajectories of alcohol consumption through early midlife, showing that frequency and quantity of alcohol use generally increase across adolescence, peak in the early or mid-20s, and decline thereafter (Britton et al., 2015; Zellers et al., 2022). Nonetheless, conducting variable-centered analyses of developmental change in indices of alcohol misuse (e.g., heavy drinking, binge drinking) remains an important next step. In the present study, we applied a variable-centered approach – multilevel growth modeling – to investigate trajectories of alcohol misuse from adolescence through early midlife in two population-based samples. In addition, we adopted a multilevel developmental contextual approach to understand individual differences in patterns in alcohol misuse over time.

## A multilevel developmental contextual approach to alcohol misuse

Windle’s multilevel developmental contextual framework proposes that substance use and problems are dynamic processes influenced by a comprehensive set of multilevel contextual factors, including person-level factors (e.g., genetic liability, personality characteristics), proximal environmental factors (e.g., features of the parent-child relationship, marital conflict), distal environmental factors (e.g., media portrayals of substance use), and related emotional, behavioral, and health problems. This framework also recognizes that person-level and environmental factors influence one another in a bidirectional manner, and the relationships between risk factors vary across development (Windle, 2010).

Consistent with the multilevel developmental contextual framework, there is evidence to suggest that genetic, person-level, and environmental factors are related to patterns of alcohol misuse across development. There is a substantial genetic component to alcohol-related outcomes (Verhulst et al., 2015), and person-level psychological factors, such as personality traits and neurocognitive functioning, have also been associated with alcohol misuse (Courtney & Polich, 2009; Kotov et al., 2010; Sher et al., 1997; Stautz & Cooper, 2013). Further, environmental factors play an important role in the development of alcohol use and problems, though the relevance of specific environmental factors varies based on developmental stage. For example, parenting behaviors and peer substance use are strongly associated with patterns of alcohol misuse in adolescence (Leung et al., 2014; Patrick & Schulenberg, 2014; Steinberg et al., 1994), whereas romantic relationship status and employment assume increasing importance in young adulthood (Boden et al., 2017; Fischer & Wiersma, 2012; Lee et al., 2015).

## Predictors of alcohol misuse in the 30s and 40s

Early midlife remains an understudied period, but several cross-sectional and prospective studies have examined predictors of alcohol misuse in the 30s and 40s. In person-centered trajectory analyses, a number of adolescent and young adult factors have been linked with increasing or consistently high trajectory groups, including alcohol and other substance use, truancy, and internalizing problems (Berg et al., 2019; Jackson & Sher, 2005; Meier et al., 2013; Virtanen et al., 2015; Warner et al., 2007). Results from prospective, variable-centered analyses lend additional support for each of these factors as predictors of early midlife alcohol outcomes (Merline et al., 2008; Schulenberg et al., 2016). Moreover, cross-sectional analyses have identified behavioral and psychosocial factors concurrently related to heavy drinking or alcohol problems in early midlife, such as other substance use and dependence, physical health, internalizing problems, employment and job demands, financial resources, and parenthood (Berg et al., 2013; Schulenberg et al., 2016). Nonetheless, correlates of *developmental change* in alcohol misuse through early midlife remain uncharacterized.

## The current study

We studied two population-based samples in order to describe trajectories of alcohol misuse across adolescence through early midlife, estimate genetic and environmental contributions to individual differences in those trajectories, and identify factors associated with both initial levels of and changes in alcohol misuse. This study builds upon previous work in several ways. First, we applied a variable-centered approach to characterize trajectories of alcohol misuse from adolescence through early midlife. Second, we used data from a longitudinal study of Finnish twins to evaluate the degree to which individual differences in trajectories of alcohol misuse are explained by additive genetic, shared environmental, and unique environmental factors. A prior meta-analysis of twin and adoption studies suggests that approximately 50% of the variation in alcohol use disorder (AUD) is explained by genetic factors, and 10% is attributable to environmental factors that are shared by co-twins (e.g., family income, parental divorce, neighborhood characteristics) (Verhulst et al., 2015). Recent longitudinal studies have further demonstrated that changes in frequency and quantity of alcohol use across adolescence and adulthood are influenced by genetic factors (Drouard et al., 2023; Zellers et al., 2022). However, genetic and environmental components of alcohol misuse trajectories from adolescence through early midlife have not been studied.

Third, we investigated adolescent and young adult correlates of alcohol misuse trajectories. Consistent with the multilevel developmental contextual approach, we included correlates from a wide range of domains, such as other substance use, physical health, educational attainment, employment, and romantic relationships. We prioritized factors that have been associated with alcohol misuse in the 30s and 40s in previous work (Berg et al., 2013, 2019; Jackson & Sher, 2005; Meier et al., 2013; Merline et al., 2008; Schulenberg et al., 2016; Virtanen et al., 2015; Warner et al., 2007). Finally, we used population-based samples from both Finland (FinnTwin16) and the United States (the National Longitudinal Study of Adolescent to Adult Health) to investigate similarities and differences in developmental trajectories of alcohol misuse across cultural contexts. Because available measures of alcohol-related outcomes differed across samples, we focused on frequency of intoxication in FinnTwin16 and frequency of binge drinking in the National Longitudinal Study of Adolescent to Adult Health (Add Health). In what follows, we refer to both measures using the term “alcohol misuse” for parsimony. Our hypotheses and analytic plan were pre-registered using the Open Science Framework (doi:10.17605/OSF.IO/9D6 WB).

## Method

### Participants

Participants were from two population-based longitudinal studies: FinnTwin16 and the National Longitudinal Study of Adolescent to Adult Health (Add Health). FinnTwin16 is a study of five consecutive birth cohorts of Finnish twins born 1975–1979 (Kaidesoja et al., 2019). Twins completed mailed surveys when they were 16 years old and were invited to participate in follow-up surveys at ages 17 (97% retention), 18 (97% retention), 25 (88% retention), and 35 (79% retention). Analyses were limited to 5,659 individuals (2,750 complete twin pairs, 159 singletons; 52% female; 32% monozygotic) who initiated alcohol use during the study period. For twin pairs who were discordant for lifetime history of alcohol use, only the co-twin who initiated alcohol use was retained in the analysis. Adolescent factors were from assessments at ages 16, 17, and 18, young adult factors were from the age 25 assessment, and the alcohol misuse outcome was measured at all five time points. Participants provided informed consent, and data were collected in accordance with Institutional Review Board and Research Ethics Committee guidelines.

Add Health is a nationally representative longitudinal study of more than 20,000 adolescents in the United States. Interviews were conducted in 1994 (Wave I), 1996 (Wave II; 76% retention), 2001–2002 (Wave III; 80% retention), 2008–2009 (Wave IV; 83% retention), and 2016–2018 (Wave V; 67% retention) (Harris et al., 2019). Participants ranged in age from 11 to 21 years at Wave I, 12–22 years at Wave II, 18–27 years at Wave III, 24–34 years at Wave IV, and 33–44 years at Wave V. The current sample was limited to individuals who initiated alcohol use during the study period (N=18,026, 50% female). Participants self-identified as White (64%), Black (21%), Native American (4%), Asian (7%), or Other race/ethnicity (9%); 17% of participants also identified as Hispanic or Latino. Adolescent factors were from the Waves I and II assessments, young adult factors were from the Waves III and IV assessments, and the alcohol misuse outcome was measured at all five time points. To ensure that Add Health was comparable to FinnTwin16 regarding developmental timing, analyses of adolescent factors were limited to participants between ages 15 and 19 at the time of assessment. Analyses of young adult factors were limited to participants between ages 20 and 29. All assessments of alcohol misuse were used in the analyses, with no exclusions based on age. Participants provided written informed consent in accordance with Institutional Review Board guidelines.

### FinnTwin16 measures

#### Alcohol misuse

Alcohol misuse was assessed in FinnTwin16 using one item: “How often do you get really drunk?” Response options were “once a week or more,” “about 1–2 times a month,” “less often than that,” and “never.” Ordinal response options were recoded as a pseudo-continuous measure of days intoxicated per month using the mid-point of each response option, such that “once a week or more” was coded as 17, “about 1–2 times a month” was coded as 1.5, “less often than that” was coded as 0.5, and “never” was coded as 0. To disambiguate correlates of alcohol misuse from correlates of alcohol initiation, individuals who indicated that they never consume alcohol were recoded as missing.

#### Adolescent correlates

Correlates of alcohol misuse trajectories measured during adolescence included age of alcohol use onset, peer alcohol use, cigarette smoking, physical health, body mass index (BMI), sleeping difficulties, and grades.

#### Age of alcohol use onset

At age 16, participants reported their age when they first drank at least a glass of beer, at least a glass of wine, a long drink, and hard liquor. The earliest age reported across these four items was recorded as the participant’s age of alcohol use onset.

#### Peer alcohol use

At ages 16, 17, and 18, participants were asked, “What proportion of your same-sex peers drink [beer, wine, liquor] every now and then?” Response options were 1 = “almost everyone,” 2 = “most,” 3 = “half,” 4 = “some,” and 5 = “hardly any.” Items were reverse-coded, and peer alcohol use was recorded as the participant’s maximum value across items and assessments.

#### Smoking

Cigarette smoking was measured at ages 16, 17, and 18 using one item: “Which of the following best describes your present smoking habits?” At age 16, response options were 1 = “I have never smoked,” 2 = “I smoke less often than once a week,” 3 = “I smoke once or more a week but not every day,” and 4 = “I smoke once or more daily.” At ages 17 and 18, the maximal response options were “I smoke every day but no more than 9 cigarettes per day” and “I smoke at least 10 cigarettes a day;” these responses options were combined to remain consistent with the age 16 assessment. Adolescent smoking was then recorded as the participant’s maximal value across assessments.

#### Overall physical health

At ages 16, 17, and 18, participants were asked whether they viewed their health as 1 = “very good,” 2 = “rather good,” 3 = “mediocre,” 4 = “rather poor,” or 5 = “very poor.” This item was reverse-coded, and the participant’s minimum value across assessments was utilized in the analysis.

#### BMI

At ages 16, 17, and 18, participants were asked to report their current height and weight. BMI was calculated as weight (in kilograms) divided by height-squared (in meters-squared). The participant’s maximum value across assessments was used in the analysis.

#### Sleeping difficulties

Sleeping difficulties at ages 16, 17, and 18 were assessed using one item: “During the past six months, how often have you had difficulty getting to sleep or staying asleep?” Response options included 1 = “seldom or never,” 2 = “about once a month,” 3 = “about once a week,” and 4 = “almost every day.” Adolescent sleeping difficulties was then recorded as the participant’s maximum value across assessments.

#### Grades

Participants reported on their grades at age 16 using one item: “What kind of grades did you receive last term? Compared to the average in your class or course, were they much better [1], somewhat better [2], about average [3], somewhat below average [4], or considerably below average [5]?” This item was reverse-coded, such that 1 = “considerably below average” and 5 = “much better” than the average.

#### Young adult correlates

Correlates measured in young adulthood included cigarette smoking, nicotine dependence, other drug use, educational attainment, employment, job satisfaction, financial status, cohabitation status, number of romantic partnerships involving cohabitation, relationship satisfaction, parenthood, psychological distress, physical activity, importance of religion, frequency of religious service attendance, satisfaction with free time, physical health, BMI, and sleeping difficulties. All young adult correlates were measured at the age 25 assessment.

#### Smoking

Smoking behavior was measured using one item: “Which of the following best describes your present smoking habits?” Response options were 1 = “I smoke at least 20 cigarettes per day,” 2 = “I smoke 10–19 cigarettes per day,” 3 = “I smoke at most 9 cigarettes a day,” 4 = “I smoke once a week or more often but not daily,” 5 = “I smoke less than once a week,” 6 = “I have quit smoking,” and 7 = “I have never smoked.” This item was reverse-coded.

#### Nicotine dependence

Participants who reported smoking at least 20 cigarettes per day were coded as meeting criteria for nicotine dependence (1), and all others were coded as 0.

#### Other drug use

Other drug use was measured using one item: “Have you ever used hash, marijuana, or other drugs or sniffed glue?” Response options were 1 = “no,” 2 = “1–3 times,” 3 = “4–9 times,” 4 = “10–19 times,” and 5 = “more than 20 times.”

#### Educational attainment

Participants reported their highest level of education as primary school (1), high school (2), trade school (3), vocational school (4), or university or vocational college of university standing (5).

#### Employment

Participants reported whether they were primarily working outside the home, working at home, a student, unemployed, in the military, or doing something else. In addition, participants recorded how many hours per week they work for wages. These measures were recoded as follows: unemployed, not in the workforce (working at home, student, or doing something else), employed part-time (working outside the home less than 39 hours per week), and employed full-time (working outside the home 40+ hours per week or in the military). A set of dummy-coded variables was created, with employed full-time as the reference category.

#### Job satisfaction

Job satisfaction was measured using one item: “Are you satisfied with your success at work and in your studies?” Response options were 1 = “completely,” 2 = “mostly,” 3 = “somewhat,” 4 = “mostly not,” and 5 = “not at all.” This item was reverse-coded.

#### Financial status

Participants indicated whether their present financial situation was 1 = “very bad,” 2 = “fairly bad,” 3 = “average,” 4 = “fairly good,” or 5 = “very good.”

#### Cohabitation

Participants were asked whether they lived with a spouse or romantic partner, their parents, alone, alone with children, or someone else. Participants who reported that they lived with a spouse or romantic partner were coded as 1. All others were coded as 0.

#### Number of romantic partnerships with cohabitation

Participants reported how many romantic relationships they had where they lived together with someone. Response options were 1 = “none,” 2 = “one,” 3 = “two,” and 4 = “three or more.”

#### Relationship satisfaction

Participants reported on the degree to which they were satisfied with their relationship with their partner using the following response options: 1 = “not at all,” 2 = “mostly not,” 3 = “somewhat,” 4 = “mostly,” and 5 = “completely.”

#### Parenthood

At age 25, participants were asked whether they had children of their own (0 = “no,” 1 = “yes”).

#### Psychological distress

Psychological distress was measured using the 20-item General Health Questionnaire (Goldberg, 1972) (α=0.92). Each item was rated on a 4-point Likert scale, with higher values indicating greater psychological distress. Among individuals with complete data on 10 or more items, psychological distress was recorded as a prorated mean score.

#### Work-related physical activity

Participants indicated whether their work was 1 = “largely sedentary work, not much walking during the working day,” 2 = “sedentary or standing work involving some walking, but not much lifting or carrying,” 3 = “working involving a lot of walking, lifting, or carrying,” or 4 = “heavy manual work, involving lifting or carrying heavy objects, digging, logging, etc.”

#### Physical activity during leisure time

Physical activity was measured using two items: “How often do you exercise in your leisure time?” and “How long do you exercise per occasion?” The product of these two items was computed as a measure of time spent exercising per month.

#### Importance of religion

Participants were asked, “How important do you think religiousness is in your life?” (1 = “very important,” 2 = “important,” 3 = “not very important,” 4 = “not at all important”). This item was reverse-coded.

#### Frequency of religious service attendance

Religious service attendance was evaluated using one item: “Not counting weddings, funerals, and baptisms, how often do you go to church or other religious events?” (1 = “once a week,” 2 = “once a month,” 3 = “once a year,” 4 = “less often,” 5 = “not at all”). This item was reverse-coded.

#### Satisfaction with free time

Participants reported on the degree to which they were satisfied with their leisure time at home and outside the home (1 = “completely,” 2 = “mostly,” 3 = “somewhat,” 4 = “mostly not,” and 5 = “not at all”). Each of these items was reverse-coded.

#### Overall physical health

Participants rated their physical health as 1 = “very poor,” 2 = “rather poor,” 3 = “mediocre,” 4 = “rather good,” or 5 = “very good.”

#### BMI

Participants reported their height and weight at age 25. BMI was calculated as weight (in kilograms) divided by height-squared (in meters-squared).

#### Sleeping difficulties

Sleeping difficulties were measured using one item: “During the past six months, how often have you had difficulty getting to sleep or staying asleep?” Response options included 1 = “seldom or never,” 2 = “about once a month,” 3 = “about once a week,” and 4 = “almost every day.”

#### Covariates

Participant sex (0 = female, 1 = male) was included as a covariate in all analyses.

### Add Health measures

#### Alcohol misuse

One item was used as an index of alcohol misuse: “Over the past 12 months, on how many days did you drink 5 or more drinks in a row?” Response options included “none,” “1 or 2 days,” “once a month or less,” “2 or 3 days a month,” “1 or 2 days a week,” “3–5 days a week,” and “every day or almost every day.” Ordinal response options were recoded as a pseudo-continuous measure of binge drinking days per month using the mid-point of each response option. Individuals who indicated that they had not consumed alcohol in the past year were coded as missing.

#### Adolescent correlates

Correlates of alcohol misuse trajectories measured during adolescence included age of alcohol use onset, peer alcohol use, cigarette smoking, physical health, BMI, and grades.

#### Age of alcohol use onset

At Waves I, II, and IV, participants reported their age when they first had a drink of beer, wine, or liquor while not with their parents.

#### Peer alcohol use

At Waves I and II, peer alcohol use was measured using one item: “Of your three best friends, how many drink alcohol at least once a month?” Responses ranged from 0 to 3. Peer alcohol use was recorded as the participant’s maximum value across assessments.

#### Smoking

Cigarette smoking was measured at Waves I and II using two items: “During the past 30 days, on how many days did you smoke cigarettes?” and “During the past 30 days, on the days you smoked, how many cigarettes did you smoke each day?” The product of these two items was computed to create a measure of cigarettes smoked per month, and adolescent smoking was recorded as the participant’s maximum value across assessments.

#### Overall physical health

At Waves I and II, participants rated their health as 1 = “excellent,” 2 = “very good,” 3 = “good,” 4 = “fair,” or 5 = “poor.” The item was reverse-coded, and the participant’s minimum value across assessments was used in the analysis.

#### BMI

Participants reported their current height and weight at Waves I and II. BMI was calculated as weight (in kilograms) divided by height-squared (in meters-squared). The participant’s maximum value across assessments was utilized in the analysis.

#### Grades

At Waves I and II, participants reported their grade in English/Language Arts, Mathematics, History/Social Studies, and Science (1 = A, 2 = B, 3 = C, 4 = D or lower). Each item was reverse-coded, and the mean was computed across subjects. The participant’s grades were then recorded as the minimum value across assessments.

### Young adult correlates

Correlates measured in young adulthood included cigarette smoking, nicotine dependence, cannabis problems, educational attainment, employment, job satisfaction, financial status, financial difficulties, number of romantic partnerships involving cohabitation, relationship satisfaction, parenthood, psychological distress, physical activity, importance of religion, frequency of religious service attendance, frequency of other religious activities, physical health, and BMI.

#### Smoking

Cigarette smoking was measured at Waves III and IV using two items: “During the past 30 days, on how many days did you smoke cigarettes?” and “During the past 30 days, on the days you smoked, how many cigarettes did you smoke each day?” The product of these two items was used as a measure of cigarettes smoked per month. Young adult smoking was then recorded as the participant’s maximum value across assessments.

#### Nicotine dependence

Nicotine dependence was assessed at Waves III and IV using the Fagerstrom Test for Nicotine Dependence (FTND) (Heatherton et al., 1991) (α=0.77 at Wave III, α=0.79 at Wave IV). The participant’s maximum FTND score was used in the analysis.

#### Cannabis problems

Number of DSM-IV cannabis use disorder symptoms was evaluated at Wave IV.

#### Educational attainment

At Wave IV, participants reported on their highest level of education: 8^th^ grade or less, some high school, high school graduate, some vocation/technical training, completed vocational/ technical training, some college, completed college, some graduate school, completed a master’s degree, some graduate training beyond a master’s degree, completed a doctoral degree, some post-baccalaureate professional education, or completed post-baccalaureate professional education.

#### Employment

Employment status at Waves III and IV was evaluated using one item: “Are you currently working for pay for at least 10 hours a week?” Participants also reported on how many hours per week they spend working for pay. These measures were recoded as follows: not in the workforce (did not endorse working for pay at least 10 hours per week), employed part-time (working 10–39 hours per week), and employed full-time (working 40+ hours per week). Dummy-coded variables were created with employed full-time as the reference category. Participants who differed in their employment status across waves were categorized as “not in the workforce” if they met these criteria at either assessment and as “employed part-time” if they were employed part-time at one assessment and employed full-time at another assessment.

#### Job satisfaction

At Waves III and IV, job satisfaction was measured using one item: “How satisfied are you with this job, as a whole?” Response options were 1 = “extremely satisfied,” 2 = “satisfied,” 3 = “neither satisfied nor dissatisfied,” 4 = “dissatisfied,” and 5 = “extremely dissatisfied.” This item was reverse-coded, and the participant’s minimum satisfaction with their job was used in the analysis.

#### Financial status

Financial status was measured at Wave IV using one item: “Suppose you were to sell all of your possessions and pay off your debts. Would you have something left over [1], break even [2], or be in debt [3]?”

#### Financial difficulties

At Waves III and IV, participants were asked whether they have been without telephone service, were unable to pay the full amount of the rent or mortgage, were evicted for not paying the rent or mortgage, did not pay the full amount of a utility bill, or had the service turned off by the gas or electric company. The participant’s maximum number of financial problems across Waves III and IV was used in the analysis.

#### Number of romantic partnerships with cohabitation

At Waves III and IV, participants were asked, “With how many people have you lived in a marriage-like relationship?” The participant’s maximum number of cohabiting partners was incorporated in the analysis.

#### Relationship satisfaction

Relationship satisfaction was measured at Wave III using one item: “In general, how satisfied are you with your relationship with your partner”? Response options were 1 = “very satisfied,” 2 = “somewhat satisfied,” 3 = “neither dissatisfied or satisfied,” 4 = “somewhat dissatisfied,” and 5 = “very dissatisfied.” This item was reverse-coded, such that higher values reflect greater relationship satisfaction.

#### Parenthood

Participants who reported at least one live birth by Waves III or IV were coded as 1, and all others were coded as 0.

#### Psychological distress

At Waves III and IV, psychological distress was assessed using 9 items from the Center for Epidemiologic Studies Depression Scale (Andresen et al., 1994) (α=0.81 at Wave III, α=0.81 at Wave IV). Each item was rated on a 4-point scale, with higher values indicating greater psychological distress. A sum score was computed, and the participant’s maximum value across assessments was used in the analysis.

#### Work-related physical activity

At Wave III, work-related physical activity was evaluated using four items, which asked how many hours per week the participant spent doing hard, moderate, and light physical work, and being seated. Based on which level of physical activity characterized their work the majority of the time, work-related physical activity was coded as 1 = seated, 2 = light physical work, 3 = moderate physical work, and 4 = hard physical work.

#### Physical activity during leisure time

Physical activity was measured at Waves III and IV using 7 items, which asked how many times the participant had participated in a series of activities (e.g., team sports, cycling, jogging) within the past week. For each assessment, a total sum score was calculated to represent the total number of times that the participant exercised in the past week; the minimum value across Waves III and IV was used in the analysis.

#### Importance of religion

Participants were asked, “How important is religion to you?” (1 = “not important,” 2 = “somewhat important,” 3 = “very important,” and 4 = “more important than anything else”). The minimum value across Waves III and IV was utilized in the analysis.

#### Frequency of religious service attendance

Participants reported on their religious service attendance using one item: “In the past 12 months, how often did you attend religious services?” (0 = “never,” 1 = “a few times,” 2 = “once a month,” 3 = “2 or 3 times a month,” 4 = “once a week,” and 5 = “more than once a week”). The minimum value across Waves III and IV was used in the analysis.

#### Frequency of other religious activities

Participants reported on their attendance at other religious activities using one item: “In the past 12 months, how often did you attend church activities outside of regular worship service?” (0 = “never,” 1 = “a few times,” 2 = “once a month,” 3 = “2 or 3 times a month,” 4 = “once a week,” and 5 = “more than once a week”). The minimum value across Waves III and IV was utilized in the analysis.

#### Overall physical health

At Waves III and IV, participants rated their health as 1 = “excellent,” 2 = “very good,” 3 = “good,” 4 = “fair,” and 5 = “poor.” This item was reverse-coded, and physical health was recorded as the participant’s minimum value across assessments.

#### BMI

Participants reported their current height and weight at Waves III and IV. BMI was calculated as weight (in kilograms) divided by height-squared (in meters-squared), and young adult BMI was recorded as the participant’s maximum value across assessments.

#### Covariates

Participant sex (0 = female, 1 = male), racial/ethnic identity, and year of assessment were included as covariates. Race/ethnicity was aggregated into the following classifications: Non-Hispanic White, Hispanic White, non-Hispanic Black, Hispanic Black, and Other. A set of dummy-coded variables was created, with non-Hispanic White serving as the reference group.

### Statistical analysis

We characterized trajectories of alcohol misuse from adolescence through early midlife using a mixed-effects growth curve model based on orthogonal variance components (McArdle, 2006). A mixed-effects growth curve model based on orthogonal variance components is written as $Y{\left[t\right]}_{\text{n}}=\text{U}0_{\text{n}}+\propto [t]\text{U}1_{\text{n}}+(\propto [t]+1)\text{C}01_{\text{n}}+U{\left[t\right]}_{\text{n}}$, where Y[t] is the outcome of interest (in this case, alcohol misuse), and ∝ represents the timing of assessments. According to the above equation, for each individual (n=1 to N), their alcohol misuse is described in terms of a unique score for alcohol misuse at baseline (U0); a unique score for linear change in alcohol misuse across the study period (U1); a score describing the covariance between their initial level of alcohol misuse and their change in alcohol misuse over time (C01), which is uncorrelated with the unique scores (U0,U1); and measurement error at each assessment (U[t]). In the full sample, initial levels of alcohol misuse and changes in alcohol misuse over time are thus described by fixed means $\left(\beta_{0},\beta_{1}\right)$, as well as random variances $\left({\sigma_{u0}}^{2},{\sigma_{u1}}^{2}\right)$ and a covariance $\left({\sigma_{c01}}^{2}\right)$ describing inter-individual variation in trajectories of alcohol misuse. The model can be further expanded to include unique and common scores for non-linear change in alcohol misuse over time.

To ensure that analyses of FinnTwin16 and Add Health were comparable, age 16 was used as the “baseline” in both samples (i.e., time was fixed to 0 at age 16). Analyses accounted for nesting of data within individuals (Add Health and FinnTwin16) and within families (FinnTwin16 only). We constructed intercept-only, linear, and quadratic growth models, then selected the best-fitting model based on the Akaike information criterion (AIC), the Bayesian information criterion (BIC), and likelihood ratio tests.

Next, we leveraged the twin design of FinnTwin16 to examine genetic and environmental contributions to trajectories of alcohol misuse. We used the longitudinal biometric variance component model (McArdle, 2006), which expands upon the mixed-effects growth curve model by describing contributions of additive genetic (A), shared environmental (S), and unique environmental (E) factors to initial levels of alcohol misuse and changes in alcohol misuse over time. The longitudinal biometric variance component model invokes the same assumptions as the classical twin design: namely, that monozygotic twins share 100% of their genes, dizygotic twins share 50% of their genes, and the degree to which twins share family and community experiences is equal for monozygotic and dizygotic twins. The unique environment includes experiences not shared by twins and random error.

Finally, to identify adolescent and young adult factors associated with trajectories of alcohol misuse, we conducted two sets of analyses: one with adolescent correlates included as predictors, and one with young adult factors included as predictors. Significant predictors (p<.05) were carried forward into a combined model to evaluate whether associations with trajectories of alcohol misuse remained significant after accounting for factors from other developmental periods. Models included the main effect of each factor, which reflects the association between the adolescent or young adult factor and initial levels of alcohol misuse, as well as an interaction term between the factor and time, which reflects the degree to which the adolescent or young adult factor was associated with change in alcohol misuse over time.

Sex was included as a covariate in analyses of both samples. Racial/ethnic identity and year of assessment were included as covariates in analyses of Add Health only because FinnTwin16 is ethnically homogeneous. All analyses were conducted using SAS PROC MIXED in SAS 9.4 (©2002–2012 SAS Institute Inc., Cary NC US).

## Results

### Descriptive statistics

Table 1 provides descriptive statistics for the study variables. Mean levels of alcohol misuse are presented by wave of assessment for ease of presentation, though age was used as the metric for time in the primary analyses. The distribution of alcohol misuse is shown separately by sex in Figures S1 and S2 for FinnTwin16 and Add Health, respectively.

In FinnTwin16, the mean value for frequency of alcohol misuse was lowest at the age 16 assessment and highest at the age 25 assessment. On average, participants reported drinking to intoxication once every 1 to 2 months (i.e., 0.77 days per month) at age 16. At age 25, participants drank to intoxication an average of 3 days per month. In Add Health, the mean value for frequency of alcohol misuse among past-year drinkers was lowest at the Wave V assessment (mean age = 37.56 years) and highest at the Wave II assessment (mean age = 16.24 years), such that, on average, participants reported binge drinking 1.84 days per month at Wave V and 2.49 days per month at Wave II.

### Trajectories of alcohol misuse

We constructed intercept-only, linear, and quadratic growth curve models to represent changes in alcohol misuse from adolescence through early midlife. As shown in Table 2, a quadratic growth curve model provided the best fit to the data in both FinnTwin16 and Add Health: The quadratic model was associated with lower AIC and BIC values than the intercept-only and linear models, and likelihood ratio tests indicated that the linear model provided significantly worse model fit when compared to the quadratic model^1^.

The quadratic growth curve model includes fixed effects, which represent the expected mean at age 16$\left(\beta_{0}\right)$, the expected linear change in alcohol misuse at age 16$\left(\beta_{1}\right)$, and how the rate of change in alcohol misuse varies per decade $\left(\beta_{2}\right)$. The model also includes random effects, which represent individual differences in alcohol misuse trajectories. Across FinnTwin16 and Add Health, there was significant inter-individual variability in initial levels of alcohol misuse at age 16 $\left({\sigma_{u0}}^{2}\right)$ and in linear $\left({\sigma_{u1}}^{2}\right)$ and non-linear $\left({\sigma_{u2}}^{2}\right)$ change in alcohol misuse over time. The intercept and linear slope showed a negative covariance $\left({\sigma_{c01}}^{2}\right)$, meaning that individuals who reported higher initial levels of alcohol misuse had a slower increase in alcohol misuse. Similarly, values for the linear and quadratic slopes were negatively related to one another $\left({\sigma_{c12}}^{2}\right)$ whereas the intercept and quadratic slope were positively related $\left({\sigma_{c02}}^{2}\right)$.

**Figure 1.**
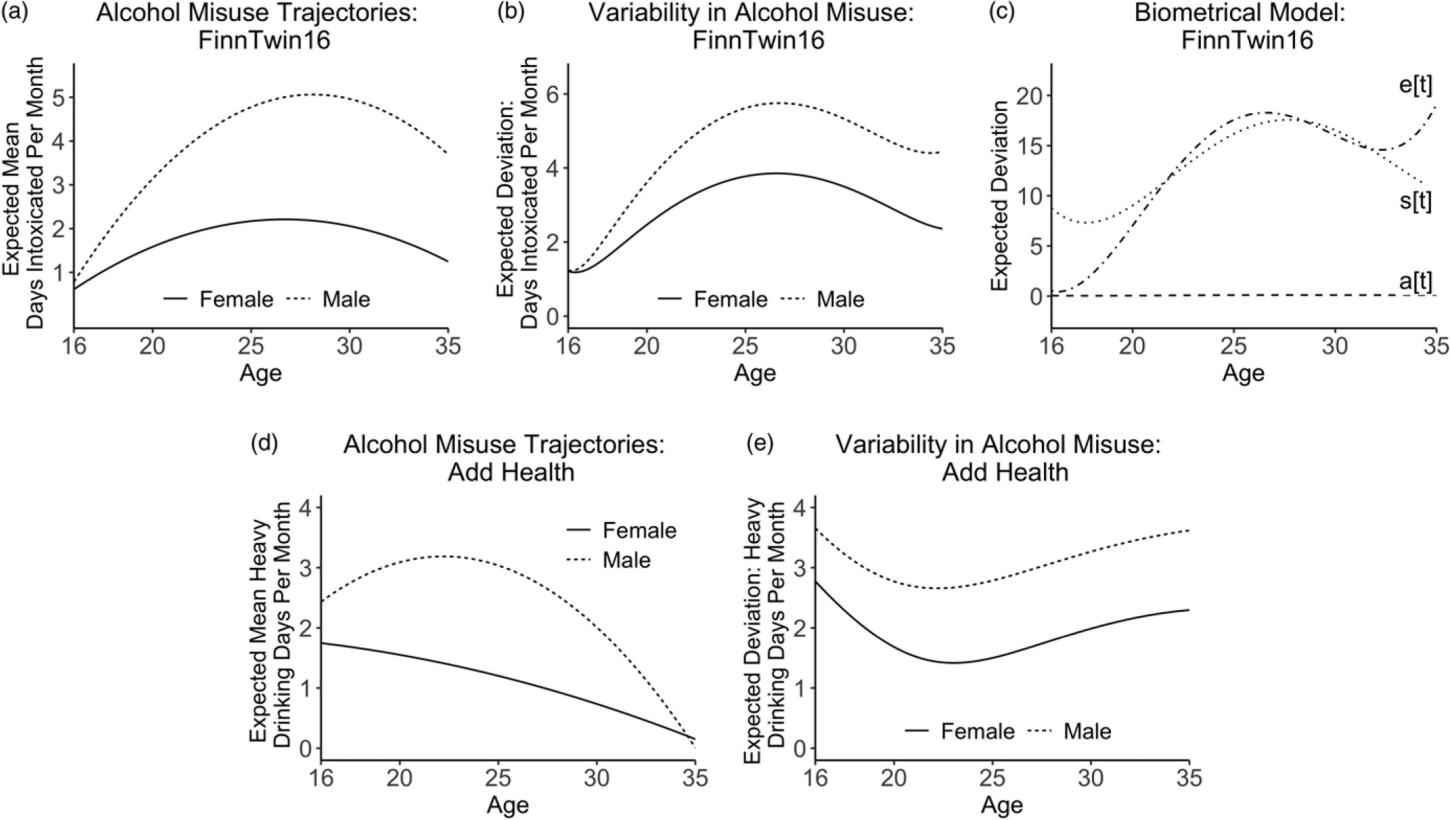
Trajectories of alcohol misuse from adolescence through early midlife. (***a***) Expected mean frequency of alcohol misuse based on the quadratic growth curve model parameters in FinnTwin16. (***b***) Expected deviation from the mean trajectory of alcohol misuse in FinnTwin16. (***c***) Expected contributions of additive genetic factors, a[t]; shared environmental factors, s[t]; and unique environmental factors, e[t], to deviation from the expected trajectory of alcohol misuse, presented as a function of age. (***d***) Expected mean frequency of alcohol misuse based on the quadratic growth curve model parameters in Add Health. (***e***) Expected deviation from the mean trajectory of alcohol misuse in Add Health.

In Figure 1, the expected frequency of alcohol misuse over time (derived from the quadratic model parameters) and expected deviation from the average trajectory of alcohol misuse are plotted separately by sex. For both males and females in FinnTwin16 (Fig. 1a) and males in Add Health (Fig. 1d), frequency of alcohol misuse increased after age 16, peaked in early adulthood, and declined thereafter. For females in Add Health, frequency of alcohol misuse declined from age 16 through age 35. In FinnTwin16, the predicted age of maximal alcohol misuse was 27 years for females and 28 years for males. Inter-individual variability in alcohol misuse also increased from age 16 into young adulthood, peaking at age 27 in both male and female participants and declining thereafter (Fig. 1b). In Add Health, maximal alcohol misuse occurred at an earlier age: 16 years for females and 22 years for males. Variability in alcohol misuse declined from age 16, reached a minimum at ages 22 and 23 in males and females, respectively, then increased across the remainder of the study period (Fig. 1e).

### Genetic and environmental components of alcohol misuse trajectories

In FinnTwin16, we applied the longitudinal biometric variance component model to describe contributions of additive genetic, shared environmental, and unique environmental factors to inter-individual variability in alcohol misuse trajectories. Model results are presented in Table 3. When combined across random effect parameters using the method described by McArdle (2006), additive genetic factors accounted for 0.3% of the total inter-individual variability in alcohol misuse trajectories from adolescence through early midlife. Shared environmental factors accounted for 38.0%, unique environmental factors accounted for 58.3%, and time-specific error accounted for 3.3% of the variance. Though the contributions of additive genetic factors were small in magnitude, only the additive genetic component of the covariance between the intercept and quadratic slope $({\sigma^{2}}_{\text{ac02}})$ could be fixed to zero without a significant decrease in model fit (Table 3).

To ease interpretation, we plotted model-implied additive genetic, shared environmental, and unique environmental contributions to deviations from the expected trajectory of alcohol misuse as a function of time (McArdle, 2006). As shown in Figure 1c, variability in alcohol misuse attributable to shared environmental factors increased from age 16 through age 28 and declined thereafter. Variation attributable to additive genetic factors remained small in magnitude across the study period.

### Adolescent and young adult correlates of alcohol misuse trajectories

**Figure 2.**
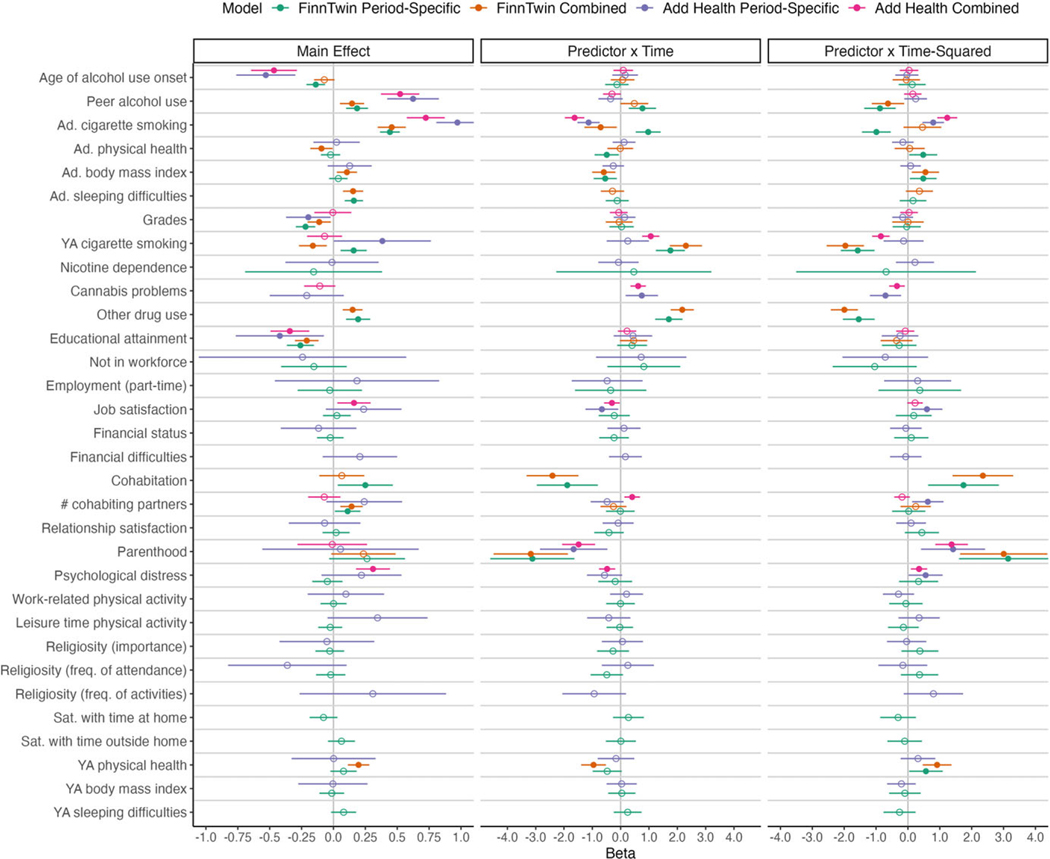
Adolescent and young adult correlates of alcohol misuse trajectories. Adolescent and young adult factors were first examined in separate models. For each factor, we estimated its relationship with frequency of alcohol misuse at age 16 (main effect) and the degree to which the variable modified linear (predictor × time) and quadratic (predictor × time-squared) change in alcohol misuse. Results from these period-specific models are shown in green for FinnTwin16 and in purple for Hdd Health. If a variable was statistically significantly (p<.05) associated with the intercept, linear slope, or quadratic slope, it was carried forward into a combined model (shown in orange for FinnTwin16 and pink for Add Health). Statistically significant (p<.05) parameter estimates are shown as a filled circle, and non-significant estimates are shown as an open circle. Across models, ordinal and continuous predictors were standardized, but the outcome was not. Therefore, the beta estimate represents the expected change in days intoxicated per month (FinnTwin16) or binge drinking days per month (Add Health) associated with a 1-standard deviation increase in the predictor. Error bars represent 95% confidence intervals. Ad = adolescent; YA = young adult; # = number; freq = frequency; sat = satisfaction.

Next, we evaluated associations of a series of adolescent and young adult factors with trajectories of alcohol misuse. In FinnTwin16 and Add Health, we constructed two initial models: The first included adolescent correlates of alcohol misuse trajectories, and the second included young adult correlates. For each adolescent and young adult factor, we estimated its relationship with frequency of alcohol misuse at age 16 and the degree to which the variable moderated linear and quadratic change in alcohol misuse over time. If a variable was statistically significantly associated with the intercept, linear slope, or quadratic slope, it was carried forward into a combined model. Results from the adolescent, young adult, and combined models are summarized in Figure 2.

#### Correlates of alcohol misuse trajectories in FinnTwin16

In FinnTwin16, the combined model included seven variables measured in adolescence age of alcohol use onset, peer alcohol use, smoking, physical health, BMI, sleeping difficulties, and grades and seven variables measured in young adulthood smoking, other drug use, educational attainment, current cohabitation, number of partnerships involving cohabitation, parenthood, and physical health. For brevity, we focus our review below on statistically significant associations.

#### Associations with initial levels of alcohol misuse

In the combined model, adolescents who reported a higher proportion of drinking peers, greater cigarette smoking, greater BMI, and more sleeping difficulties indicated more frequent alcohol misuse at age 16. On the other hand, adolescents with better physical health and higher grades exhibited less frequent alcohol misuse. Lower levels of smoking, more frequent use of other drugs, lower educational attainment, more romantic partnerships involving cohabitation, and better physical health at age 25 were also associated with higher initial levels of alcohol misuse.

#### Associations with change in alcohol misuse over time

**Figure 3.**
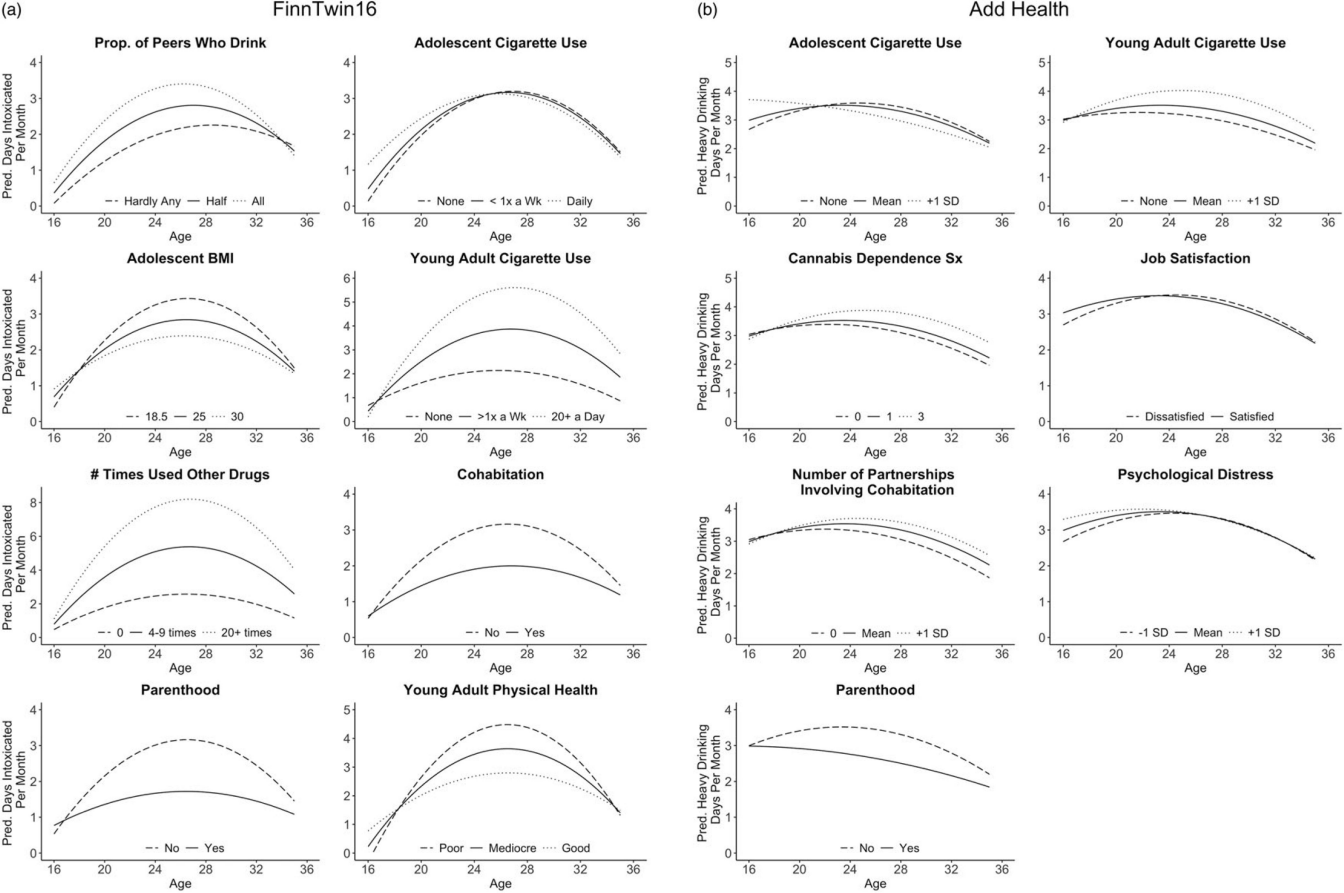
Predicted frequency of alcohol misuse as a function of adolescent and young adult predictors. (***a***) peer alcohol use, adolescent cigarette smoking, adolescent body mass index, young adult cigarette smoking, other drug use, cohabitation, parenthood, and young adult physical health were associated with linear and/or non-linear change in alcohol misuse in FinnTwin16. Trajectories of alcohol misuse are presented across levels of each of these predictors. (***b***) adolescent and young adult cigarette smoking, cannabis dependence symptoms, job satisfaction, number of romantic partnerships involving cohabitation, psychological distress, and parenthood were associated with linear and/or non-linear change in alcohol misuse in Add Health. Trajectories of alcohol misuse are presented across levels of each of these predictors. Pred = predicted; prop = proportion; < 1x a wk = less than one time per week; BMI = body mass index; > 1x a wk = more than one time per week; SD = standard deviation; sx = symptoms.

The interactions of adolescent and young adult smoking, adolescent BMI, other drug use, cohabitation, parenthood, and young adult physical health with time were statistically significant, suggesting that these factors were associated with linear change in alcohol misuse. In addition, peer alcohol use, adolescent BMI, young adult smoking, other drug use, cohabitation, parenthood, and young adult physical health were related to quadratic change in alcohol misuse. The model-implied trajectories of alcohol misuse across levels of these variables are presented in Figure 3a.

#### Correlates of alcohol misuse trajectories in Add Health

In Add Health, the combined model included four variables measured in adolescence age of alcohol use onset, peer alcohol use, smoking, and grades and seven variables measured in young adulthood smoking, cannabis problems, educational attainment, job satisfaction, number of romantic partnerships involving cohabitation, parenthood, and psychological distress.

#### Associations with initial levels of alcohol misuse

In the combined model, adolescents who reported an earlier age of alcohol use onset, affiliated with a greater number of drinking peers, and endorsed greater cigarette smoking reported more frequent alcohol misuse at age 16. Lower educational attainment, greater job satisfaction, and higher young adult psychological distress were also related to greater alcohol misuse at age 16.

#### Associations with change in alcohol misuse over time

The interactions of adolescent and young adult smoking, cannabis problems, job satisfaction, number of romantic partnerships involving cohabitation, psychological distress, and parenthood with time were statistically significant, suggesting that these factors were related to linear change in alcohol misuse. Further, adolescent smoking, cannabis problems, psychological distress, and parenthood were related to quadratic change in alcohol misuse. For each variable associated with linear or non-linear change in alcohol misuse, the model-implied trajectories are presented in Figure 3b.

## Discussion

Our aims were to (1) characterize trajectories of alcohol misuse from adolescence through early midlife, (2) describe genetic and environmental contributions to individual differences in those trajectories, (3) identify adolescent and young adult factors associated with initial levels of alcohol misuse and changes in alcohol misuse over time, and (4) compare and contrast findings across two population-based cohorts. Consistent with prior work on rates of alcohol misuse across the lifespan (Jackson & Sartor, 2016; Lee & Sher, 2018), the expected trajectory of alcohol misuse from adolescence through early midlife generally followed an inverted U-shaped curve: Frequency of alcohol misuse increased after age 16, peaked in early adulthood, and declined thereafter. Nonetheless, we also observed substantial inter-individual variability in trajectories of alcohol misuse, which was primarily attributable to environmental factors.

Overall, the developmental course of alcohol misuse was similar across FinnTwin16, a population-based sample of Finnish twins, and Add Health, a population-based sample from the United States, with two exceptions. First, while the expected trajectory of alcohol misuse followed an inverted U-shaped curve across sex in FinnTwin16, frequency of alcohol misuse declined across the study period in female individuals in Add Health. This pattern of results may be driven by our decision to code past-year nondrinkers as missing. As a result, only 16-year-olds who report past-year alcohol use (a relatively high-risk group) contributed to the predicted value for frequency of alcohol misuse at age 16, potentially increasing the estimated intercept. To evaluate this possibility, we repeated the analyses with past-year nondrinkers coded as zero. In these supplementary analyses, the predicted frequency of alcohol misuse at age 16 was lower, and the expected inverted U-shaped curve was observed across sex and samples (Figure S3). Furthermore, and as expected, when past-year nondrinkers were coded as zero, the predicted age at peak alcohol misuse was 27 years for females and 28 years for males in FinnTwin16, and 20 years for females and 23 years for males in Add Health.

This highlights another notable difference in the pattern of results across samples: The age at peak alcohol misuse was substantially earlier in Add Health when compared to FinnTwin16. One potential explanation is that escalation of binge drinking during the college years, which is reliably observed in the United States (Schulenberg & Patrick, 2011), may contribute to an earlier age of maximal alcohol misuse: Indeed, prior studies of drinking patterns in the United States have similarly noted that the prevalence and frequency of binge drinking peaks in the early 20s (Patrick et al., 2016, 2019). Another non-mutually exclusive explanation is that role transitions associated with reductions in alcohol misuse (e.g., marriage, parenthood) generally occur later in Finland than in the United States (National Center for Health Statistics, 2016; Statistics Finland, 2020).

Next, in FinnTwin16 only, we estimated the contributions of genetic and environmental factors to individual differences in alcohol misuse trajectories. It was surprising that additive genetic factors accounted for a very small (but statistically significant) proportion of the variance, particularly in view of substantial evidence that AUD is moderately heritable (Verhulst et al., 2015). Prior cross-sectional analyses in FinnTwin16 have also supported genetic influences on alcohol misuse (Dick et al., 2011; Penninkilampi-Kerola et al., 2005; Viken et al., 1999). Of note, additive genetic factors accounted for little variance in the unique component of the intercept in the present analyses. However, this parameter is not directly comparable to parameter estimates from cross-sectional univariate twin models because the biometric growth curve model accounts for the correlations among growth factors, partitioning the variance into components that are unique to a specific growth factor and shared between growth factors. Additive genetic, shared environmental, and unique environmental contributions to each of those variance components are then estimated.

Therefore, to replicate the cross-sectional findings from previous work, we conducted cross-sectional univariate twin models to investigate the etiology of alcohol misuse at each assessment. We fitted the full univariate ACE model, then tested simpler models without shared environmental influences (AE model) and without additive genetic influences (CE model). At age 16, the full ACE model provided the best fit to the data (Table S1). Approximately 47%, 28%, and 25% of the variance in age 16 alcohol misuse was attributable to additive genetic, shared environmental, and unique environmental factors, respectively (Table S2). For the remaining assessments, shared environmental influences on alcohol misuse could be removed without a significant change in model fit (Table S1), and the estimated heritability of alcohol misuse ranged from 59% to 77% (Table S2).

Thus, although our finding that genetic factors account for little variance in trajectories of alcohol misuse was surprising, it echoes a broader point from the developmental literature, which is that cross-sectional snapshots do not necessarily capture longitudinal change (Di Biase et al., 2023). For instance, a number of studies have shown minimal contributions of the shared environment to alcohol use and problems in adulthood (Dick, 2011; van Beek et al., 2012), and we replicated this finding in our own cross-sectional analyses (see above). Conversely, our analyses of longitudinal *change* in alcohol misuse through early midlife suggest that shared environmental influences contribute considerably to the overall shape of alcohol misuse trajectories: Perhaps features of the shared environment that are relevant to adolescent drinking behavior, such as alcohol availability in the home (Komro et al., 2007), parental divorce (Salvatore et al., 2023), or local community-level factors (Kristjansson et al., 2020), have lasting effects on an individual’s overall course of alcohol misuse but are less relevant to cross-sectional variation in alcohol misuse among adults. Nonetheless, these findings warrant replication in other samples.

Finally, we investigated whether a series of adolescent and young adult factors were related to alcohol misuse frequency at age 16 and to changes in alcohol misuse over time. We focus on three findings that were observed across both samples. First, substance-related variables were associated with alcohol misuse trajectories in both FinnTwin16 and Add Health. Adolescent cigarette smoking was related to alcohol misuse at age 16 and to changes in alcohol misuse over time. Moreover, higher levels of smoking and other drug use in young adulthood were associated with a steeper increase in alcohol misuse and higher predicted frequency of alcohol misuse across the study period. For example, among individuals who used illicit drugs 20 or more times in FinnTwin16, the predicted frequency of alcohol misuse at age 35 was 4 days intoxicated per month (compared to 1 day per month among individuals who never used other drugs). These findings are consistent with prior longitudinal studies (Chassin et al., 2002; Merline et al., 2008; Schulenberg et al., 2016) and underscore the potential long-term impact of efforts to reduce substance use among adolescents and young adults (Griffin & Botvin, 2010; White & Rabiner, 2012).

Second, individuals who were parents by young adulthood exhibited less frequent alcohol misuse than those who remained childless. The negative association between parenthood and alcohol misuse is in line with recent work (Rose et al., 2022) and with role compatibility theory, which suggests that individuals with high levels of substance use may delay marriage or parenthood (role selection) and that adopting a social role incompatible with substance-related behaviors leads to reduced substance use (role socialization) (Yamaguchi & Kandel, 1985). Third, we did not find evidence that employment and financial status in young adulthood were related to trajectories of alcohol misuse, which contrasts with prior cross-sectional studies of heavy drinking and AUD in early midlife (Berg et al., 2013; Schulenberg et al., 2016). Though job loss and financial difficulties may cooccur with alcohol misuse and problems, they did not modify the developmental course of alcohol misuse in the present study.

### Limitations

These findings should be considered in view of several limitations. First, alcohol misuse was assessed using only one item, and sample-based differences in the measurement of alcohol misuse (frequency of intoxication in FinnTwin16, frequency of binge drinking in Add Health) may contribute to differences in the pattern of results. Second, drinking to intoxication is not included in other definitions of alcohol misuse (National Institute on Alcohol Abuse and Alcoholism, n.d.), which focus on binge and heavy drinking. However, a study of perceived drunkenness among Finnish adolescents found that girls and boys who reported moderate drunkenness had consumed, on average, 5 and 6 drinks, respectively (Lintonen et al., 2004), suggesting that drinking to intoxication may be a reasonable indicator of alcohol misuse. Third, due to data availability, our analyses only considered frequency of intoxication (FinnTwin16) and frequency of binge drinking (Add Health) as indicators of alcohol misuse. Therefore, it will be important for future research to consider other dimensions of alcohol misuse (e.g., heavy drinking, high-intensity drinking), as well as measures of clinically significant alcohol problems. The pattern and correlates of alcohol trajectories are likely to differ based on the alcohol-related outcome under study: Prior work suggests that indices of alcohol consumption, misuse, and problems have an overlapping, but partially distinct, genetic architecture (Dick et al., 2011; Kranzler et al., 2019), and different predictors emerge in analyses of heavy drinking versus AUD (Merline et al., 2008). As a result, the findings observed in the present study may or may not generalize to other alcohol-related outcomes.

Fourth, our findings do not provide evidence for causality. For example, when considering the negative association between parenthood and change in alcohol misuse, we cannot distinguish whether becoming a parent is associated with reductions in alcohol misuse or individuals with more frequent alcohol misuse are less likely to become parents. Additional work is needed to differentiate causes and consequences of alcohol misuse. Fifth, analyses focused on specific patterns of alcohol consumption but did not control for participants’ overall alcohol intake.

## Conclusions

On average, frequency of alcohol misuse increases across adolescence, peaks in young adulthood, and declines thereafter. Even so, individuals show varying trajectories of alcohol misuse across development. As hypothesized by the multilevel developmental contextual framework, heterogeneity in the course of alcohol misuse is related to person-level, proximal environmental, and distal environmental factors. For example, one’s own substance use, physical health, and mental health (person-level factors), as well as peer substance use, cohabitation, and parenthood (proximal environments), moderated trajectories of alcohol misuse in the present study. Efforts to reduce alcohol misuse and problems in early midlife may consider these factors as the basis for selective intervention or as modifiable intervention targets. Distal environmental factors also played a role: The average age at peak alcohol misuse varied across samples from Finland and the United States, highlighting the importance of considering the broader cultural context when determining the optimal timing of prevention efforts. Ultimately, our findings underscore that alcohol misuse is a highly complex, developmentally dynamic process. Efforts to describe and reduce alcohol misuse in early midlife must consider this inherent complexity.

## Supplementary Material

1

## Figures and Tables

**Table 1.** Descriptive statistics for the primary study variables

	FinnTwin16			Add Health		
	M / %	SD	Range	M / %	SD	Range

Any past–year alcohol use T1	81.2%	–	–	53.7%	–	–

Any past–year alcohol use T2	86.3%	–	–	49.2%	–	–

Any past–year alcohol use T3	93.1%	–	–	78.4%	–	–

Any past–year alcohol use T4	95.8%	–	–	77.9%	–	–

Any past–year alcohol use T5	95.4%	–	–	88.4%	–	–

Alcohol misuse T1	0.77	2.32	0–17	2.02	5.07	0–30

Alcohol misuse T2	1.17	3.08	0–17	2.49	5.69	0–30

Alcohol misuse T3	1.56	3.78	0–17	2.12	4.57	0–30

Alcohol misuse T4	3.35	5.76	0–17	2.05	4.74	0–30

Alcohol misuse T5	2.28	4.85	0–17	1.84	5.00	0–30

Age of alcohol use onset (years)	13.48	1.39	10–16	15.82	2.81	10–33

Peer alcohol use	4.20	1.02	1–5	1.60	1.19	0–3

Adolescent cigarette smoking	2.16	1.33	1–4	68.92	157.07	0–622

Adolescent physical health	3.85	0.76	1–5	3.70	0.92	1–5

Adolescent BMI (kg/m^2^)	21.46	2.38	13.87–29.13	22.40	4.08	9.65–35.25

Adolescent sleeping difficulties	1.95	1.00	1–4	–	–	–

Grades	3.44	1.01	1–5	2.68	0.75	1–4

Cigarette smoking	2.79	2.03	1–7	117.28	210.49	0–796

Nicotine dependence	3.2%	–	–	1.10	1.86	0–9

Cannabis problems	–	–	–	0.89	1.72	0–7

Other drug use	1.42	0.95	1–5	–	–	–

Educational attainment	3.27	1.18	1–5	5.73	2.18	1–13

Employment (unemployed)	7.8%	–	–	–	–	–

Employment (not in workforce)	45.9%	–	–	32.8%	–	–

Employment (part-time)	19.1%	–	–	23.4%	–	–

Job satisfaction	3.73	0.96	1–5	3.74	0.96	1–5

Financial status	3.13	0.91	1–5	1.63	0.83	1–3

Financial difficulties	–	–	–	0.53	0.98	0–5

Current cohabitation	46.6%	–	–	–	–	–

# of cohabiting relationships	1.67	0.69	1–4	0.83	0.95	0–4

Relationship satisfaction	4.06	1.14	1–5	4.48	0.92	1–5

Parenthood	12.4%	–	–	50.5%	–	–

Psychological distress	1.87	0.44	1.00–4.00	5.62	4.23	0–18

Work-related physical activity	1.92	1.03	1–4	2.35	1.16	1–4

Physical activity	13.66	12.96	0–60	4.78	4.98	0–20

Importance of religion	2.01	0.83	1–4	1.34	0.86	0–3

Freq. of relig. service attendance	1.94	1.04	1–5	1.28	1.41	0–5

Freq. of other relig. activities	–	–	–	0.33	0.89	0–5

Sat. with free time at home	3.93	0.80	1–5	–	–	–

Sat. with free time outside home	3.91	0.81	1–5	–	–	–

Young adult physical health	4.13	0.72	1–5	3.70	0.92	1–5

Young adult BMI (kg/m^2^)	22.94	3.24	15.62–33.30	28.32	6.86	13.79–49.83

Young adult sleeping difficulties	1.86	0.96	1–4	–	–	–

*Note.* Descriptive statistics are not always comparable across samples, as FinnTwin16 and Add Health often used different measures of the same construct. See the Method section for additional details. In FinnTwin16, age of alcohol use onset was only measured at the baseline assessment (i.e., at age 16). As a result, age 16 is the maximum age of alcohol use onset shown in the Table, though some individuals may have initiated alcohol use at a later age. T1 = time 1 (age 16 assessment in FinnTwin16; Wave I assessment in Add Health); T2 = time 2 (age 17 assessment in FinnTwin16; Wave II assessment in Add Health); T3 = time 3 (age 18 assessment in FinnTwin16; Wave III assessment in Add Health); T4 = time 4 (age 25 assessment in FinnTwin16; Wave IV assessment in Add Health); T5 = time 5 (age 35 assessment in FinnTwin16; Wave V assessment in Add Health); BMI = body mass index; freq = frequency; relig = religious; sat = satisfaction.

**Table 2.** Parameter estimates from phenotypic mixed-effects growth curve models of alcohol misuse

	FinnTwin16			Add Health		
	Intercept-Only Model	Linear Model	Quadratic Model	Intercept-Only Model	Linear Model	Quadratic Model
Parameter	Est. [95% CI]	Est. [95% CI]	Est. [95% CI]	Est. [95% CI]	Est. [95% CI]	Est. [95% CI]

$\beta_{0}$	1.78 [1.71, 1.85]	1.24 [1.17, 1.31]	0.70 [0.63, 0.76]	2.21 [2.13, 2.29]	2.21 [2.10, 2.31]	2.12 [1.99, 2.25]

$\beta_{1}$	–	1.05 [0.96, 1.14]	4.87 [4.52, 5.22]	–	0.47 [0.07, 0.87]	1.88 [1.24, 2.53]

$\beta_{2}$	–	–	−4.20 [−4.56, −3.85]	–	–	−3.68 [−4.83, −2.53]

${\sigma_{u0}}^{2}$	3.29 [3.03, 3.56]	−0.58 [−0.99, −0.18]	2.44 [2.03, 2.85]	5.27 [4.95, 5.59]	11.4 [10.5, 12.3]	16.1 [14.8, 17.4]

${\sigma_{u1}}^{2}$	–	1.65 [1.09, 2.21]	183.0 [169.8, 196.2]	–	5.80 [5.11, 6.50]	39.6 [31.3, 47.9]

${\sigma_{u2}}^{2}$	–	–	176.0 [163.6, 188.4]	–	–	9.58 [4.70, 14.5]

${\sigma_{c\text{0}1}}^{2}$	–	1.79 [1.55, 2.03]	−1.88 [−2.87, −0.90]	–	−2.88 [−3.27, −2.50]	−10.7 [−12.2, −9.15]

${\sigma_{c\text{0}2}}^{2}$	–	–	1.42 [0.45, 2.39]	–	–	5.86 [4.69, 7.02]

${\sigma_{c12}}^{2}$	–	–	−88.5 [−94.9, −82.2]	–	–	−10.0 [−13.2, −6.88]

${\sigma_{u}}^{2}$	14.3 [14.0, 14.6]	11.8 [11.5, 12.1]	7.39 [7.17, 7.60]	19.5 [19.2, 19.9]	17.2 [16.8, 17.6]	16.6 [16.2, 17.0]

Model χ^*2*^	χ^*2*^(1) = 1047.12, *p* < .0001	χ^*2*^(3) = 2571.29, *p* < .0001	χ^*2*^(6) = 5035.47, *p* < .0001	χ^*2*^(1) = 1484.15, *p* < .0001	χ^*2*^(3) = 1854.44, *p* < .0001	χ^*2*^(6) = 2087.31, *p* < .0001

AIC	125843.4	123854.1	120789.0	245805.6	245440.8	245178.6

BIC	125863.3	123893.9	120855.4	245836.7	245503.1	245279.8

LRT	–	χ^*2*^(2) = 1995.3, *p* < .0001	χ^*2*^(3) = 3073.1, *p* < .0001	–	χ^*2*^(2) = 372.80, *p* < .0001	χ^*2*^(3) = 272.20, *p* < .0001

Est = estimate; CI = confidence interval; AIC = Akaike Information Criterion; BIC = Bayesian Information Criterion; LRT = likelihood ratio test.

**Table 3.** Parameter estimates from the biometric mixed−effects growth curve model of alcohol misuse in FinnTwin16

Parameter	Est. [95% CI]	Likelihood ratio test (parameter is dropped)
$\beta_{0}$	–0.97 [–1.10, –0.84]	–
$\beta_{1}$	4.10 [3.64, 4.55]	–
$\beta_{2}$	–4.72 [–5.17, –4.28]	–
${{\text{σ}}^{2}}_{\text{eu}0}$	0.47 [–0.22, 1.16]	–
${{\text{σ}}^{2}}_{\text{suo}}$	12.1 [11.0, 13.1]	*χ*^2^(1) = 735.20, *p* < .0001
${{\text{σ}}^{2}}_{\text{auo}}$	0.07 [0.03, 0.10]	*χ*^2^(1) = 25.10, *p* < .0001
${{\text{σ}}^{2}}_{\text{eu1}}$	165.8 [145.0, 186.6]	–
${{\text{σ}}^{2}}_{\text{su1}}$	119.5 [100.0, 139.0]	*χ*^2^(1) = 158.70, *p* < .0001
${{\text{σ}}^{2}}_{\text{au1}}$	0.95 [0.37, 1.52]	*χ*^2^(1) = 19.40, *p* < .0001
${{\text{σ}}^{2}}_{\text{eu2}}$	172.0 [152.4, 191.5]	–
${{\text{σ}}^{2}}_{\text{su2}}$	88.6 [71.3, 106.0]	*χ*^2^(1) = 107.40, *p* < .0001
${{\text{σ}}^{2}}_{\text{au2}}$	0.79 [0.29, 1.28]	*χ*^2^(1) = 18.00, *p* < .0001
${{\text{σ}}^{2}}_{\text{ec}01}$	–2.55 [–4.18, –0.93]	–
${{\text{σ}}^{2}}_{sc0}$	–9.18 [–11.1, –7.21]	*χ*^2^(1) = 94.80, *p* < .0001
${{\text{σ}}^{2}}_{\text{ac}01}$	–0.07 [–0.14, –0.01]	*χ*^2^(1) = 49.10, *p* < .0001
${{\text{σ}}^{2}}_{\text{ec}02}$	2.61 [0.99, 4.23]	–
${{\text{σ}}^{2}}_{sc02}$	5.85 [3.97, 7.73]	*χ*^2^(1) = 39.90, *p* < .0001
${{\text{σ}}^{2}}_{\text{ac}02}$	0.05 [–0.01, 0.11]	*χ*^2^(1) = 3.10, *p* = .0783
${{\text{σ}}^{2}}_{\text{ec}12}$	–83.0 {–93.0, –73.0]	–
${{\text{σ}}^{2}}_{\text{sc}12}$	–50.3 [–59.4, –41.2]	*χ*^2^(1) = 127.20, *p* < .0001
${{\text{σ}}^{2}}_{\text{ac}12}$	–0.42 [–0.69, –0.16]	*χ*^2^(1) = 60.10, *p* < .0001
${{\text{σ}}^{2}}_{u}$	14.3 [13.9, 14.7]	–
Model Chi–Square	*χ*^2^ (18) = 6729.14, *p* < .0001	–
AIC	172307.2	–
BIC	172438.7	–

*Note.* Random variances with the subscript u0 represent variability in U0, a unique score for alcohol misuse at baseline. The subscript u1 refers to variability in U1, a unique score for linear change in alcohol misuse, and the subscript u2 refers to variability in U2, a unique score for quadratic change in alcohol misuse. The subscript c01 refers to the covariance between initial levels and linear change in alcohol misuse over time; c02 and c12 refer to the covariances between the intercept and quadratic slope, and between the linear and quadratic slopes, respectively. Contributions of additive genetic, shared environmental, and unique environmental factors to each of these random variance parameters are denoted by the subscripts a, s, and e, respectively. ${{\text{σ}}^{2}}_{u}$ represents time−specific measurement error. Est = estimate; CI = confidence interval; AIC = Akaike Information Criterion; BIC = Bayesian Information Criterion.

## Footnotes

1 As suggested by a reviewer, we also examined piecewise linear models in both samples, with the knot point fixed at age 22. This model did not converge in Add Health. In FinnTwin16, the quadratic model provided better fit to the data than the piecewise model based on AIC (120789.0 versus 120895.5) and BIC (120855.4 versus 120975.6). Therefore, we proceeded with results from the quadratic growth model.

## References

[R1] Andresen EM, Malmgren JA, Carter WB, & Patrick DL (1994). Screening for depression in well older adults: Evaluation of a short form of the CES-D (Center for epidemiologic studies depression scale). American Journal of Preventive Medicine, 10(2), 77–84. 8037935

[R2] Berg N, Kiviruusu O, Karvonen S, Kestilä L, Lintonen T, Rahkonen O, & Huurre T. (2013). A 26-year follow-up study of heavy drinking trajectories from adolescence to mid-adulthood and adult disadvantage. Alcohol and Alcoholism, 48(4), 452–457. 10.1093/alcalc/agt026 23531717

[R3] Berg N, Kiviruusu OH, Lintonen TP, & Huurre TM (2019). Longitudinal prospective associations between psychological symptoms and heavy episodic drinking from adolescence to midlife. Scandinavian Journal of Public Health, 47(4), 420–427. 10.1177/1403494818769174 29644935

[R4] Boden JM, Lee JO, Horwood LJ, Grest CV, & McLeod GFH (2017). Modelling possible causality in the associations between unemployment, cannabis use, and alcohol misuse. Social Science & Medicine, 175, 127–134. 10.1016/j.socscimed.2017.01.001. 28088618

[R5] Brady J. (2006). The association between alcohol misuse and suicidal behaviour. Alcohol and Alcoholism, 41(5), 473–478. 10.1093/alcalc/agl060 16891335

[R6] Britton A, Ben-Shlomo Y, Benzeval M, Kuh D, & Bell S. (2015). Life course trajectories of alcohol consumption in the United Kingdom using longitudinal data from nine cohort studies. BMC Medicine, 13(1), 47. 10.1186/s12916-015-0273-z 25858476 PMC4351673

[R7] Case A, & Deaton A. (2015). Rising morbidity and mortality in midlife among white non-hispanic Americans in the 21st century. Proceedings of The National Academy of Sciences of The United States of America, 112(49), 15078–15083. 10.1073/pnas.1518393112 26575631 PMC4679063

[R8] Chassin L, Pitts SC, & Prost J. (2002). Binge drinking trajectories from adolescence to emerging adulthood in a high-risk sample: Predictors and substance abuse outcomes. Journal of Consulting and Clinical Psychology, 70(1), 67–78. 10.1037/0022-006X.70.1.67 11860058

[R9] Cho Y, Shin S-Y, Won S, Relton CL, Davey Smith G, & Shin M-J (2015). Alcohol intake and cardiovascular risk factors: A Mendelian randomisation study. Scientific Reports, 5(1), 18422. 10.1038/srep18422 26687910 PMC4685310

[R10] Courtney KE, & Polich J. (2009). Binge drinking in young adults: Data, definitions, and determinants. Psychological Bulletin, 135(1), 142–156. 10.1037/a0014414 19210057 PMC2748736

[R11] Di Biase MA, Tian YE, Bethlehem RAI, Seidlitz J, Alexander-Bloch AF, Yeo BTT, & Zalesky A. (2023). Mapping human brain charts cross-sectionally and longitudinally. Proceedings of The National Academy of Sciences of The United States of America, 120(20), e2216798120. 10.1073/pnas.2216798120 PMC10193972 37155868

[R12] Dick DM (2011). Developmental changes in genetic influences on alcohol use and dependence. Child Development Perspectives, 5(4), 223–230. 10.1111/j.1750-8606.2011.00207.x

[R13] Dick DM, Meyers JL, Rose RJ, Kaprio J, & Kendler KS (2011). Measures of current alcohol consumption and problems: Two independent twin studies suggest a complex genetic architecture. Alcoholism: Clinical and Experimental Research, 35(12), 2152–2161. 10.1111/j.1530-0277.2011.01564.x 21689117 PMC3215847

[R14] Drouard G, Silventoinen K, Latvala A, & Kaprio J. (2023). Genetic and environmental factors underlying parallel changes in body mass index and alcohol consumption: A 36-year longitudinal study of adult twins. Obesity Facts, 16(3), 224–236. 10.1159/000529835 36882010 PMC10826601

[R15] Fischer JL, & Wiersma JD (2012). Romantic relationships and alcohol use. Current Drug Abuse Reviews, 5(2), 98–116. 22455505 10.2174/1874473711205020098

[R16] Goldberg DP (1972). The detection of psychiatric illness by questionnaire: A technique for the identification and assessment of non-psychotic psychiatric illness (pp. 156). Oxford University Press.

[R17] Griffin KW, & Botvin GJ (2010). Evidence-based interventions for preventing substance use disorders in adolescents. Child and Adolescent Psychiatric Clinics of North America, 19(3), 505–526. 10.1016/j.chc.2010.03.005 20682218 PMC2916744

[R18] Harris KM, Halpern CT, Whitsel EA, Hussey JM, Killeya-Jones LA, Tabor J, & Dean SC (2019). Cohort profile: The national longitudinal study of adolescent to adult health (Add health). International Journal of Epidemiology, 48(5), 1415–1415k. 10.1093/ije/dyz115 31257425 PMC6857761

[R19] Heatherton TF, Kozlowski LT, Frecker RC, & Fagerström KO (1991). The fagerström test for nicotine dependence: A revision of the fagerström tolerance questionnaire. British Journal of Addiction, 86(9), 1119–1127. 10.1111/j.1360-0443.1991.tb01879.x 1932883

[R20] Holmes MV, Dale CE, Zuccolo L, Silverwood RJ, Guo Y, Ye Z, Prieto-Merino D, Dehghan A, Trompet S, Wong A, Cavadino A, Drogan D, Padmanabhan S, Li S, Yesupriya A, Leusink M, Sundstrom J, Hubacek JA, Pikhart H, …, & on behalf of The InterAct Consortium (2014). Association between alcohol and cardiovascular disease: Mendelian randomisation analysis based on individual participant data. BMJ, 349(jul10 6), g4164–g4164. 10.1136/bmj.g4164 25011450 PMC4091648

[R21] Hublin C, & Kaprio J. (2022). Chronotype and mortality—A 37-year follow-up study in Finnish adults. medRxiv. 10.1101/2022.04.02.22273342 37322846

[R22] Jackson KM, & Sher KJ (2005). Similarities and differences of longitudinal phenotypes across alternate indices of alcohol involvement: A methodologic comparison of trajectory approaches. Psychology of Addictive Behaviors : Journal of the Society of Psychologists in Addictive Behaviors, 19(4), 339–351. 10.1037/0893-164X.19.4.339 PMC2898721 16366806

[R23] Jackson SE, & Sartor CE (2016). The natural course of substance use and dependence. In The Oxford handbook of substance use and substance use disorders (pp. 67–134). Oxford University Press.

[R24] Jung T, & Wickrama Ka S. (2008). An introduction to latent class growth analysis and growth mixture modeling. Social and Personality Psychology Compass, 2(1), 302–317. 10.1111/j.1751-9004.2007.00054.x

[R25] Kaidesoja M, Aaltonen S, Bogl LH, Heikkilä K, Kaartinen S, Kujala UM, Kärkkäinen U, Masip G, Mustelin L, Palviainen T, Pietiläinen KH, Rottensteiner M, Sipilä PN, Rose RJ, Keski-Rahkonen A, & Kaprio J. (2019). FinnTwin16: A longitudinal study from age 16 of a population-based finnish twin cohort. Twin Research and Human Genetics, 22(6), 530–539. 10.1017/thg.2019.106 31796134

[R26] Komro KA, Maldonado-Molina MM, Tobler AL, Bonds JR, & Muller KE (2007). Effects of home access and availability of alcohol on young adolescents’ alcohol use. Addiction, 102(10), 1597–1608. 10.1111/j.1360-0443.2007.01941.x 17854336

[R27] Kotov R, Gamez W, Schmidt F, & Watson D. (2010). Linking “big” personality traits to anxiety, depressive, and substance use disorders: A meta-analysis. Psychological Bulletin, 136(5), 768–821. 10.1037/a0020327 20804236

[R28] Kranzler HR, Zhou H, Kember RL, Vickers Smith R, Justice AC, Damrauer S, Tsao PS, Klarin D, Baras A, Reid J, Overton J, Rader DJ, Cheng Z, Tate JP, Becker WC, Concato J, Xu K, Polimanti R, Zhao H, & Gelernter J. (2019). Genome-wide association study of alcohol consumption and use disorder in 274,424 individuals from multiple populations. Nature Communications, 10(1), 1–11. 10.1038/s41467-019-09480-8 PMC6445072 30940813

[R29] Kristjansson AL, Mann MJ, Sigfusson J, Thorisdottir IE, Allegrante JP, & Sigfusdottir ID (2020). Development and guiding principles of the Icelandic model for preventing adolescent substance use. Health Promotion Practice, 21(1), 62–69. 10.1177/1524839919849032 31162978 PMC6918020

[R30] Lee JO, Hill KG, Hartigan LA, Boden JM, Guttmannova K, Kosterman R, Bailey JA, & Catalano RF (2015). Unemployment and substance use problems among young adults: Does childhood low socioeconomic status exacerbate the effect? Social Science & Medicine, 143, 36–44. 10.1016/j.socscimed.2015.08.0161982. 26342911 PMC4601938

[R31] Lee MR, & Sher KJ (2018). Maturing out” of binge and problem drinking. Alcohol Research : Current Reviews, 39(1), 31–42. 30557146 10.35946/arcr.v39.1.06 PMC6104962

[R32] Leung RK, Toumbourou JW, & Hemphill SA (2014). The effect of peer influence and selection processes on adolescent alcohol use: A systematic review of longitudinal studies. Health Psychology Review, 8(4), 426–457. 10.1080/17437199.2011.587961 25211209

[R33] Lintonen T, Ahlstrom S, & Metso L. (2004). The reliability of self-reported drinking in adolescence. Alcohol and Alcoholism, 39(4), 362–368. 10.1093/alcalc/agh071 15208172

[R34] McArdle JJ (2006). Latent curve analyses of longitudinal twin data using a mixed-effects biometric approach. Twin Research and Human Genetics, 9(3), 343–359. 10.1375/twin.9.3.343 16790145

[R35] Meier MH, Caspi A, Houts R, Slutske WS, Harrington H, Jackson KM, Belsky DW, Poulton R, & Moffitt TE (2013). Prospective developmental subtypes of alcohol dependence from age 18 to 32 years: Implications for nosology, etiology, and intervention. Development and Psychopathology, 25(3), 785–800. 10.1017/S0954579413000175 23880392 PMC3725643

[R36] Merline A, Jager J, & Schulenberg JE (2008). Adolescent risk factors for adult alcohol use and abuse: Stability and change of predictive value across early and middle adulthood. Addiction, 103(s1), 84–99. 10.1111/j.1360-0443.2008.02178.x 18426542 PMC2649657

[R37] Muthén B, & Muthén LK (2000). Integrating person-centered and variable-centered analyses: Growth mixture modeling with latent trajectory classes. Alcoholism, Clinical and Experimental Research, 24(6), 882–891. 10888079

[R38] National Center for Health Statistics. (2016). Mean age of mothers is on the rise: United States, 2000–2014. Centers for Disease Control and Prevention. https://www.cdc.gov/nchs/products/databriefs/db232.htm

[R39] National Institute on Alcohol Abuse and Alcoholism (n.d.). What is alcohol misuse? Rethinking drinking. https://www.rethinkingdrinking.niaaa.nih.gov/how-much-is-too-much/Whats-the-harm/What-is-Alcohol-Misuse.aspx.

[R40] Patrick ME, & Schulenberg JE (2014). Prevalence and predictors of adolescent alcohol use and binge drinking in the United States. Alcohol Research : Current Reviews, 35(2), 193–200. 10.35946/arcr.v35.2.10 PMC3908711 24881328

[R41] Patrick ME, Terry-McElrath YM, Kloska DD, & Schulenberg JE (2016). High-intensity drinking among young adults in the United States: Prevalence, frequency, and developmental change. Alcoholism: Clinical and Experimental Research, 40(9), 1905–1912. 10.1111/acer.13164 27488575 PMC5008981

[R42] Patrick ME, Terry-McElrath YM, Lanza ST, Jager J, Schulenberg JE, & O’Malley PM (2019). Shifting age of peak binge drinking prevalence: Historical changes in normative trajectories among young adults aged 18 to 30. Alcoholism: Clinical and Experimental Research, 43(2), 287–298. 10.1111/acer.13933 30645773 PMC6432634

[R43] Penninkilampi-Kerola V, Kaprio J, Moilanen I, & Rose RJ (2005). Co-twin dependence modifies heritability of abstinence and alcohol use: A population-based study of finnish twins. Twin Research and Human Genetics, 8(3), 232–244. 10.1375/1832427054253095 15989750

[R44] Plunk AD, Syed-Mohammed H, Cavazos-Rehg P, Bierut LJ, & Grucza RA (2014). Alcohol consumption, heavy drinking, and mortality: Rethinking the J-shaped curve. Alcoholism: Clinical and Experimental Research, 38(2), 471–478. 10.1111/acer.12250 24033586 PMC3872245

[R45] Rehm J, Taylor B, Mohapatra S, Irving H, Baliunas D, Patra J, & Roerecke M. (2010). Alcohol as a risk factor for liver cirrhosis: A systematic review and meta-analysis. Drug and Alcohol Review, 29(4), 437–445. 10.1111/j.1465-3362.2009.00153.x 20636661

[R46] Roerecke M, & Rehm J. (2010). Irregular heavy drinking occasions and risk of ischemic heart disease: A systematic review and meta-analysis. American Journal of Epidemiology, 171(6), 633–644. 10.1093/aje/kwp451 20142394

[R47] Rose RJ, Latvala A, Silventoinen K, & Kaprio J. (2022). Alcohol consumption at age 18–25 and number of children at a 33-year follow-up: Individual and within-pair analyses of finnish twins. Alcoholism, Clinical and Experimental Research, 46(8), 1552–1564. 10.1111/acer.14886 35719054 PMC9545724

[R48] Salvatore JE, Aggen SH, & Kendler KS (2023). Parental divorce and trajectories of alcohol consumption in men: A genetically informative perspective. Journal of Studies On Alcohol and Drugs, 84(6), 902–912. 10.15288/jsad.23-00033 37306369 PMC10765972

[R49] Schulenberg JE, & Patrick ME (2011). Historical and developmental patterns of alcohol and drug use among college students: Framing the problem. In College drinking and drug use (pp. 13–35). Guilford Press.

[R50] Schulenberg JE, Patrick ME, Kloska DD, Maslowsky J, Maggs JL, & O’Malley PM (2016). Substance use disorder in early midlife: A national prospective study on health and well-being correlates and long-term predictors. Substance Abuse: Research and Treatment, 9(Suppl 1), 41–57. 10.4137/SART.S31437 27257384 PMC4881872

[R51] Sher KJ, Jackson KM, & Steinley D. (2011). Alcohol use trajectories and the ubiquitous cat’s cradle: Cause for concern? Journal of Abnormal Psychology, 120(2), 322–335. 10.1037/a0021813 21319874 PMC3091989

[R52] Sher KJ, Martin ED, Wood PK, & Rutledge PC (1997). Alcohol use disorders and neuropsychological functioning in first-year undergraduates. Experimental and Clinical Psychopharmacology, 5(3), 304–315. 10.1037//1064-1297.5.3.304 9260079

[R53] Statistics Finland (2020). Official statistics of Finland: Live births. Statistics Finland. https://stat.fi/tup/tilastotietokannat/index_en.html.

[R54] Stautz K, & Cooper A. (2013). Impulsivity-related personality traits and adolescent alcohol use: A meta-analytic review. Clinical Psychology Review, 33(4), 574–592. 10.1016/j.cpr.2013.03.003 23563081

[R55] Steinberg L, Fletcher A, & Darling N. (1994). Parental monitoring and peer influences on adolescent substance use. Pediatrics, 93(6 Pt 2), 1060–1064. 8197008

[R56] Substance Abuse and Mental Health Services Administration (2018). National survey on drug use and health 2018. Substance Abuse and Mental Health Services Administration. https://datafiles.samhsa.gov/.

[R57] Topiwala A, Wang C, Ebmeier KP, Burgess S, Bell S, Levey DF, Zhou H, McCracken C, Roca-Fernández A, Petersen SE, Raman B, Husain M, Gelernter J, Miller KL, Smith SM, Nichols TE, & Sachdev PS (2022). Associations between moderate alcohol consumption, brain iron, and cognition in UK biobank participants: Observational and mendelian randomization analyses. PLoS Medicine, 19(7), e1004039. 10.1371/journal.pmed.1004039 PMC9282660 35834561

[R58] Vachon DD, Krueger RF, Irons DE, Iacono WG, & McGue M. (2017). Are alcohol trajectories a useful way of identifying at-risk youth? A multiwave longitudinal-epidemiologic study. Journal of the American Academy of Child and Adolescent Psychiatry, 56(6), 498–505. 10.1016/j.jaac.2017.03.016 28545755 PMC5477663

[R59] van Beek JHDA, Kendler KS, de Moor MHM, Geels LM, Bartels M, Vink JM, van den Berg SM, Willemsen G, & Boomsma DI (2012). Stable genetic effects on symptoms of alcohol abuse and dependence from adolescence into early adulthood. Behavior Genetics, 42(1), 40–56. 10.1007/s10519-011-9488-8 21818662 PMC3253297

[R60] Verhulst B, Neale MC, & Kendler KS (2015). The heritability of alcohol use disorders: A meta-analysis of twin and adoption studies. Psychological Medicine, 45(5), 1061–1072. 10.1017/S0033291714002165 25171596 PMC4345133

[R61] Viken RJ, Kaprio J, Koskenvuo M, & Rose RJ (1999). Longitudinal analyses of the determinants of drinking and of drinking to intoxication in adolescent twins. Behavior Genetics, 29(6), 455–461. 10.1023/A:1021631122461 10857250

[R62] Virtanen P, Nummi T, Lintonen T, Westerlund H, Hägglöf B, & Hammarström A. (2015). Mental health in adolescence as determinant of alcohol consumption trajectories in the northern swedish cohort. International Journal of Public Health, 60(3), 335–342. 10.1007/s00038-015-0651-5 25609507

[R63] Warner LA, White HR, & Johnson V. (2007). Alcohol initiation experiences and family history of alcoholism as predictors of problem-drinking trajectories. Journal of Studies On Alcohol and Drugs, 68(1), 56–65. 10.15288/jsad.2007.68.56 17149518

[R64] White HR, & Rabiner DL (2012). College drinking and drug use. Guilford Press.

[R65] Windle M. (2010). A multilevel developmental contextual approach to substance use and addiction. BioSocieties, 5(1), 124–136. 10.1057/biosoc.2009.9 22754585 PMC3384512

[R66] Wood AM, Kaptoge S, Butterworth AS, Willeit P, Warnakula S, Bolton T, Paige E, Paul DS, Sweeting M, Burgess S, Bell S, Astle W, Stevens D, Koulman A, Selmer RM, Verschuren WMM, Sato S, Njølstad I, Woodward, :: : , & Danesh J. (2018). Risk thresholds for alcohol consumption: Combined analysis of individual-participant data for 599,912 current drinkers in 83 prospective studies. The Lancet, 391(10129), 1513–1523. 10.1016/S0140-6736(18)30134-X PMC5899998 29676281

[R67] Yamaguchi K, & Kandel DB (1985). On the resolution of role incompatibility: A life event history analysis of family roles and marijuana use. American Journal of Sociology, 90(6), 1284–1325. 10.1086/228211

[R68] Zellers SM, Iacono WG, McGue M, & Vrieze S. (2022). Developmental and etiological patterns of substance use from adolescence to middle age: A longitudinal twin study. Drug and Alcohol Dependence, 233, 109378. 10.1016/j.drugalcdep.2022.109378 PMC8957537 35248999

