# Supplementary material for: Patterns and predictors of alcohol misuse trajectories from adolescence through early midlife
Source: Dev Psychopathol. Author manuscript; Available in PMC 2025 Nov 1. (PMC11387953; doi:10.1017/S0954579424000543)
Supplement: 1 [file NIHMS1967728-supplement-1.docx]

**Supplemental Material:** Patterns and predictors of alcohol misuse trajectories from adolescence through early midlife


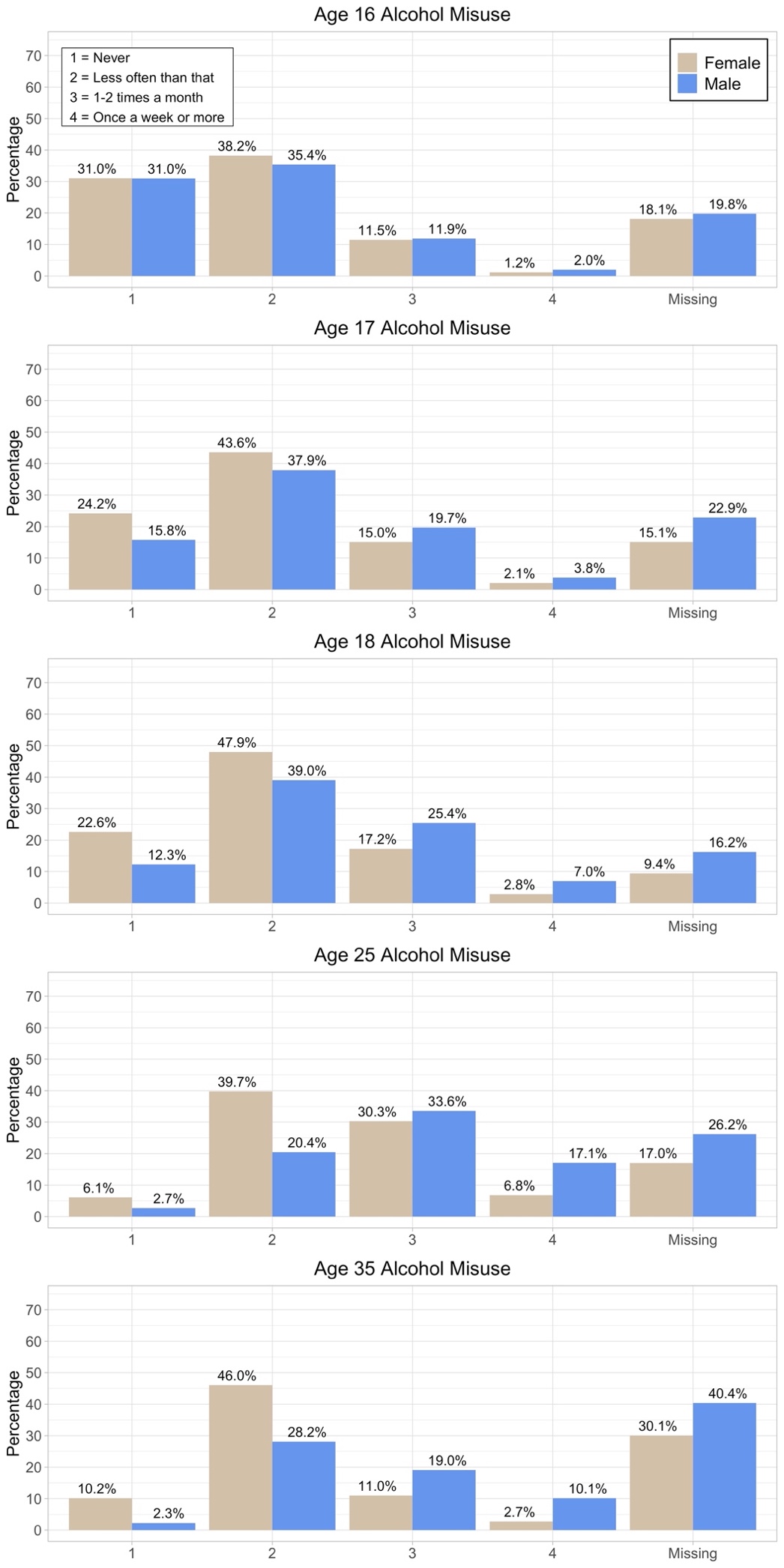


Figure S1. Distribution of drinking to intoxication in FinnTwin16, presented separately by sex. Individuals who were not current drinkers were coded as missing. Missingness also includes survey and item non-response.


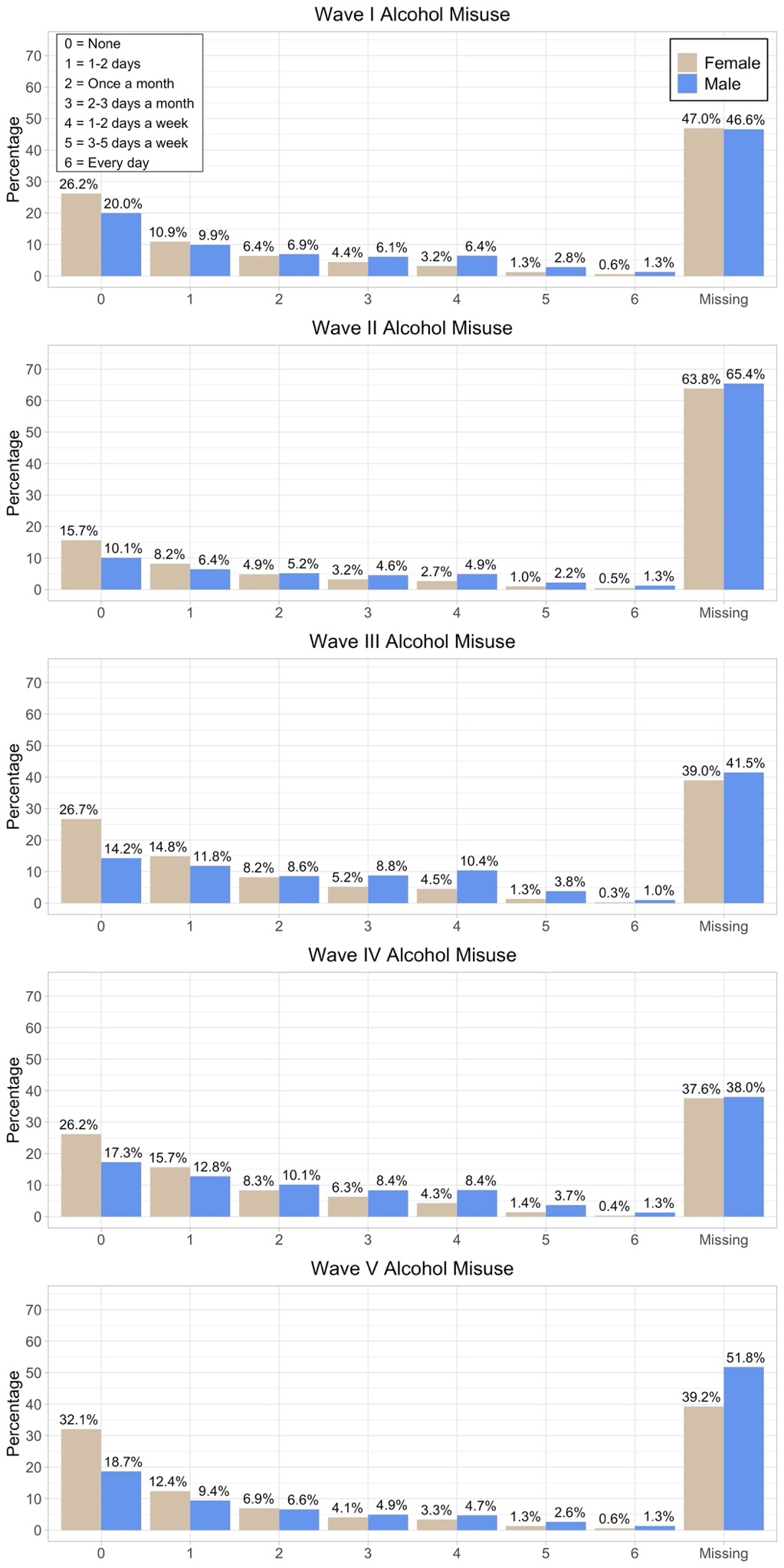


Figure S2. Distribution of binge drinking frequency in Add Health, presented separately by sex. Individuals who had not consumed alcohol in the past year were coded as missing. Missingness also includes survey and item non-response.


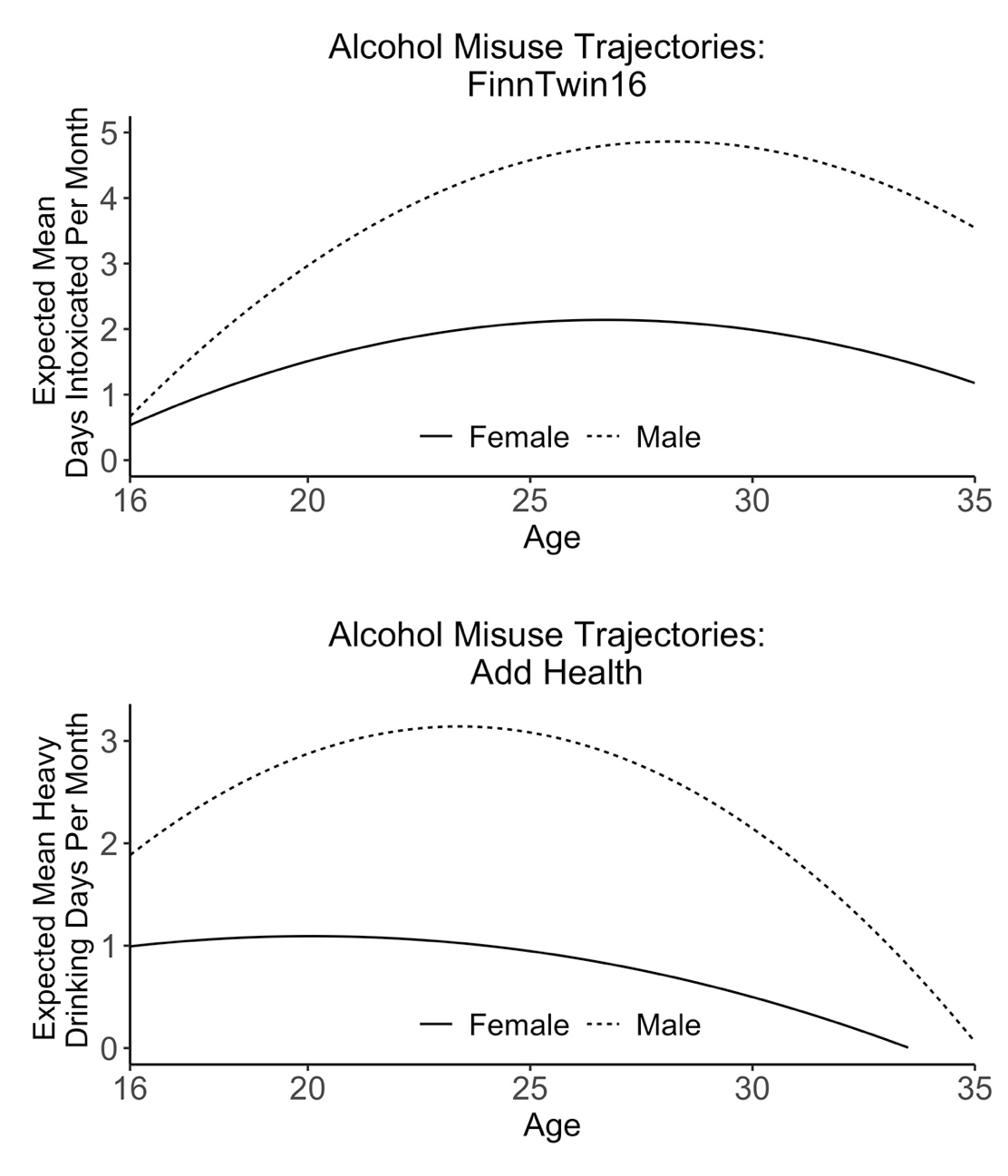


Figure S3. Expected mean frequency of alcohol misuse based on quadratic growth model parameters in FinnTwin16 and Add Health. In this supplementary analysis, past-year non-drinkers were coded as zero instead of missing.

Table S1. Univariate ACE models for alcohol misuse.

| Model | Comparison model | EP | -2LL | df | AIC | Δ-2LL | Δdf | p | |
| --- | --- | --- | --- | --- | --- | --- | --- | --- | --- |
| **Age 16** |  |  |  |  |  |  |  |  | |
| 1. ACE model | N.A. | 5 | 8883.11 | 4407 | 8893.11 | N.A. | N.A. | N.A. | |
| 2. AE model | 1 | 4 | 8907.06 | 4408 | 8915.06 | 23.95 | 1 | < .0001 | |
| 3. CE model | 1 | 4 | 8927.04 | 4408 | 8935.04 | 43.93 | 1 | < .0001 | |
| **Age 17** | | | | | | | | |  |
| 1. ACE model | N.A. | 5 | 9515.38 | 4429 | 9525.38 | N.A. | N.A. | N.A. | |
| 2. AE model | 1 | 4 | 9517.85 | 4430 | 9525.85 | 2.48 | 1 | .116 | |
| 3. CE model | 1 | 4 | 9600.13 | 4430 | 9608.13 | 84.75 | 1 | < .0001 | |
| **Age 18** | | | | | | | | |  |
| 1. ACE model | N.A. | 5 | 10662.31 | 4760 | 10672.31 | N.A. | N.A. | N.A. | |
| 2. AE model | 1 | 4 | 10663.12 | 4761 | 10671.12 | 0.81 | 1 | .367 | |
| 3. CE model | 1 | 4 | 10772.10 | 4761 | 10780.10 | 109.79 | 1 | < .0001 | |
| **Age 25** | | | | | | | | |  |
| 1. ACE model | N.A. | 5 | 9876.86 | 4298 | 9886.86 | N.A. | N.A. | N.A. | |
| 2. AE model | 1 | 4 | 9877.89 | 4299 | 9885.89 | 1.03 | 1 | .310 | |
| 3. CE model | 1 | 4 | 9931.18 | 4299 | 9939.18 | 54.33 | 1 | < .0001 | |
| **Age 35** | | | | | | | | |  |
| 1. ACE model | N.A. | 5 | 7722.57 | 3554 | 7732.57 | N.A. | N.A. | N.A. | |
| 2. AE model | 1 | 4 | 7723.78 | 3555 | 7731.78 | 1.20 | 1 | .273 | |
| 3. CE model | 1 | 4 | 7761.61 | 3555 | 7769.61 | 39.034 | 1 | < .0001 | |

*Note.* Analyses were conducted in OpenMx. Comparison model refers to the model number against which the Δ-2LL test was performed. *Abbreviations.* EP = number of estimated parameters; LL = log likelihood; df = degrees of freedom; AIC = Akaike information criterion; A = additive genetic influences; C = shared environmental influences; E = unique environmental influences; N.A. = not applicable.

Table S2. Standardized variance estimates from the best-fitting models of alcohol misuse.

|  | **A** | **C** | **E** |
| --- | --- | --- | --- |
| Age 16 | 0.47 [0.34, 0.60] | 0.28 [0.17, 0.39] | 0.25 [0.21, 0.30] |
| Age 17 | 0.77 [0.73, 0.81] | --- | 0.23 [0.19, 0.27] |
| Age 18 | 0.73 [0.68, 0.77] | --- | 0.27 [0.24, 0.32] |
| Age 25 | 0.59 [0.53, 0.64] | --- | 0.41 [0.36, 0.47] |
| Age 35 | 0.60 [0.53, 0.66] | --- | 0.40 [0.34, 0.47] |

*Note.* Estimates represent the proportion of total variance explained by additive genetic (A), shared environmental (C), and unique environmental (E) influences. Likelihood-based 95% confidence intervals are presented in brackets.
